# Research Landscape on Atherosclerotic Cardiovascular Disease and Inflammation: A Bibliometric and Visualized Study

**DOI:** 10.31083/j.rcm2309317

**Published:** 2022-09-14

**Authors:** Wende Tian, Tai Zhang, Xinyi Wang, Jie Zhang, Jianqing Ju, Hao Xu

**Affiliations:** ^1^Xiyuan Hospital, China Academy of Chinese Medical Sciences, 100091 Beijing, China; ^2^Graduate School, China Academy of Chinese Medical Sciences, 100700 Beijing, China; ^3^National Clinical Research Center for Chinese Medicine Cardiology, Xiyuan Hospital, China Academy of Chinese Medical Sciences, 100091 Beijing, China; ^4^Department of Gastroenterology, Xiyuan Hospital, China Academy of Chinese Medical Sciences, 100091 Beijing, China; ^5^Graduate School, Beijing University of Chinese Medicine, 100029 Beijing, China

**Keywords:** atherosclerotic cardiovascular disease, inflammation, bibliometrics, hotspots

## Abstract

**Background:**

The atherosclerotic cardiovascular disease (ASCVD) is a major killer and health care burden worldwide. Atherosclerosis, the common pathological foundation, has been associated with inflammation over the past few years. Some promising results also have emerged suggesting the role of targeting inflammation as a potential therapeutic option to reduce cardiovascular events. In light of the pathogenic role that inflammation plays in ASCVD, we propose to evaluate the worldwide research architecture for ASCVD and inflammation using bibliometric analysis.

**Methods:**

A search of the Web of Science Core Collection of Clarivate Analytics was performed for articles in the field published between 2012 and 2022. The number of publications per year has been visualized using GraphPad Prism through time. CiteSpace and VOSviewer were used to generate knowledge maps about the collaboration of countries, institutions, and authors, and to represent the landscape on ASCVD and inflammation research as well as to reveal current foci.

**Results:**

There were a total of 19,053 publications examined in this study. The most publications came from China (6232, 32.71%). Capital Med Univ was the most productive institution (410, 2.15%). Christian Weber published the greatest number of articles (75, 0.39%). *PloS one* was identified as the most prolific journal (706, 3.71%). *Circulation* was the most co-cited journal (13276, 2.81%). Keywords with the ongoing strong citation bursts were “nucleotide-binding oligomerization (NOD), Leucine-rich repeat (LRR)-containing protein (NLRP3) inflammasome”, “intestinal microbiota”, “exosome”, “lncRNAs”, etc.

**Conclusions:**

It can be shown that ASCVD and inflammation research benefited from manuscripts that had a high impact on the scientific community. Asian, European and North American countries dominated in the field in terms of quantitative, qualitative and collaborative parameters. The NLRP3 inflammasome, gut microbiota and trimethylamine N-oxide, autophagy, lncRNAs, exosomes, and nuclear factor erythroid 2-related factor 2 were described to be hot themes in the field.

## 1. Introduction

Atherosclerotic cardiovascular disease (ASCVD) puts patients at high risk for cardiovascular events [1], and recurrent cardiovascular events are more likely in those who have had a cardiovascular event within the past twelve months [2, 3, 4, 5, 6]. Clinically, ASCVD is defined as having the acute coronary syndrome (ACS), myocardial infarction, stable or unstable angina, coronary artery disease or arterial revascularization, ischemic stroke, transient ischemic attacks, or peripheral arterial disease including aortic aneurysm, all of atherosclerotic origin [7].

As the leading cause of death in industrialized countries and death worldwide, ASCVD accounts for approximately 650,000 deaths in the USA and 17.8 million worldwide each year [8, 9, 10]. It is estimated that ASCVD is responsible for between 33–40% of all-cause mortality among adults in the USA and EU in 2008, with a direct and indirect cost of $297.7 billion and €196 billion, respectively [11]. An estimated 35 million people experience an acute coronary event or a cerebrovascular event every year, and 25% of these people are diagnosed with ASCVD [12]. With the aging of the population blunting the benefits of improved treatments and mitigating risk factors for ASCVD, there could be a sustained and high global mortality rate by 2030 as a consequence [13].

Inflammation is in principle a coordinated response induced by tissue damage or by other stimuli to remove the initial source of cell injury, eliminate necrotic cells and tissue damage, and cause tissue repair and restore tissue homeostasis [14]. Dysregulated, excessive, or persistent inflammation, however, is damaging and is often linked to chronic conditions such as cardiovascular disorders [15]. Associated with ASCVD and related complications is atherosclerosis, an inflammatory disease. Despite cholesterol’s discovery, but not yet its relationship with atherosclerosis, Joseph Hodgson published a monograph on vascular disease in 1815 in which he blamed inflammation for causing atherosclerosis [16, 17]. In 1858, a report by Rudolf Virchow [18] described inflammatory cells in vascular plaques, and Sir William Osler suggested in 1908 that inflammation and infection play a role in the pathogenesis of atherosclerosis [19]. Despite this, the inflammation hypothesis had been disregarded for nearly a century, when atherosclerosis was believed to be caused by high blood cholesterol levels. A growing body of evidence revealed the role of inflammation in atherogenic processes by the end of the twentieth century, when the cholesterol hypothesis was challenged, and *The New England journal of medicine* published Russell Ross’s blunt description of atherosclerosis as an inflammatory disorder in 1999 [20]; since then, atherosclerosis is considered an inflammatory disease and several studies have been performed on this matter.

The pathology of atherosclerosis is characterized by persistent inflammation and failure to resolve the inflammation. In actual fact, by introducing atherogenic (apoB 100-containing) lipoproteins into the subendothelial space, an inflammatory milieu, which contributes to leukocyte recruitment and the elevated production of cytokines and interleukins, is maintained [21, 22]. As well, to maintain plaque formation and growth, other inflammation-causing cells such as T cells, mast cells, and dendritic cells contribute by enhancing cytokine production and signaling involving interferon-γ and tumor necrosis factor-α (TNF-α) [23, 24]. In the plaque’s necrotic core are lipid-laden macrophages and vascular smooth muscle cells (VSMCs), along with leukocytes resulting from failed macrophage efferocytosis [25].

Further, as athero-thrombosis evolves, inflammation also plays a critical role at multiple stages. Atherosclerosis is notorious for its thrombotic complications, which are dreaded and dramatic [26]. In the past few decades, a great deal of interest has been focused on thrombosis triggered by the rupture of the protective fibrous cap of a plaque [27]. Plaques that are vulnerable to rupture exhibit a thin fibrous cap, a large lipid-filled necrotic core, and continuous inflammation (typically manifested by macrophage infiltration) [28, 29].

Overall, the quality of the cap overlying the lipid core influences the risk of plaque rupture. A ruptured plaque occurs when the interplay between cap strength and local plaque stress is disrupted. The local plaque stress is a result of the high fibrous cap stiffness and the high endothelial shear stress [30, 31]. In a postmortem study of 113 male cadavers, it was found that most ruptured plaques had a thin fibrous cap with a mean thickness of 23 μm and 95% with a thickness of <64 μm [32]. Thin-cap fibroatheroma is therefore referred to as the specific histopathological description of this rupture-prone plaque [33, 34].

In this case, the fibrous cap that overlies the lipid core of the plaque is fractured or fissured. Fibrous cap disruption is associated with the activation of adaptive immunity [35]. Mechanistically, plaques are stabilized by SMCs and collagen. The transforming growth factor-β secreted by regulatory T (Treg) cells stimulates the maturation of extracellular collagen and positively influences collagen synthesis by SMCs [36, 37]. Collagen that is mature is responsible for providing mechanical strength to the fibrous cap, while interferon‑γ produced by activated T-helper 1 lymphocytes is a powerful destabilizing agent that inhibits SMC differentiation and proliferation as well as collagen production and maturation [36, 38, 39]. In addition, matrix metalloproteinases deteriorate collagen fibers and promote plaque vulnerability [40, 41]. Plaque ruptures are commonly found with mast cells, which release proteases that are responsible for degrading the matrix and activating matrix metalloproteinases [42]. Among cellular sources of metalloproteinases in plaques, macrophages, SMCs, and endothelial cells (ECs) are also important [40].

A ruptured atherosclerotic plaque entraps platelets that adhere to the disrupted endothelium. In turn, the platelet glycoprotein (GP) Ibα receptor interacts with both the A1 domains of von Willebrand factor (vWF), anchored to collagen by its A3 domain, and to P-selectin, for further interplay between GPIbα and vWF and the formation of thrombus [43, 44]. Activation and aggregation of platelets through the collagen receptors GPIa/IIa as well as GPVI also contribute to the development of platelet-rich thrombus [44, 45]. Furthermore, the coagulation cascade is triggered when blood coagulation proteins gain access to the atherosclerotic plaque tissue factor (TF), mainly expressed in foam cells and lipid-laden VSMCs, which leads to fibrin cross-linking and thrombus stabilization [43]. There is continual recruitment of platelets, triggered by locally accumulating mediators including thromboxane A2, adenosine diphosphate (ADP), which interacts with G-coupled-protein purinoreceptors (P2Y1 and P2Y12), and thrombin. Through activation of protease activated receptor (PAR)-1 and PAR-4, thrombin induces several G protein-coupled signaling pathways, resulting in alteration of platelets’ shape, release of dense granule contents, production of TXA2, accumulation of GP IIb/IIIa, and thrombin production, as well as the subsequent thrombotic coronary occlusion [44].

Also, pathology studies indicated that fibrous plaques without rupture may contribute to thrombotic events. It is likely that elevated low-density lipoprotein (LDL) levels facilitate the formation of atheroma and contribute to plaque disruption in general. In an era of substantial lipid lowering, the decline in the lipid content and the increase in fibrous tissue within plaques, which renders these lesions lipid-poor, making erosion a viable contribution to the residual burden of ACS in patients with plaques, despite highly intensive lipid treatment [46, 47].

It is still unclear precisely what the typical microstructural characteristics of eroded plaques are; however, well recognized features include an absence of endothelial lining overlying a plaque rich in SMCs and extracellular matrix components, especially hyaluronan, with smaller lipid and necrotic cores, fewer macrophages, and less inflammation as compared to ruptured plaques [48]. Therefore, plaque erosion exhibits an intact and thick fibrous cap and involves a discontinuity in the intimal endothelial lining; in eroded plaques, a platelet-rich white thrombus that develops does not interact with the plaque’s core.

The underlying pathophysiology of plaque erosion is not well understood even though it is identified as an alternative mechanism for thrombosis and a significant cause of sudden death [49]; however, this is likely to involve a distinct pathophysiology. It is of note that the combination of multifactorial mechanisms, such as dysregulated hyaluronan cleavage, endothelial dysfunction, toll-like receptor (TLR) signaling, leukocyte activation, and modification of sub-endothelial matrix by ECs or SMCs, may lead to loss of adhesion to the extracellular matrix or endothelial apoptosis, causing erosion [50].

In plaque erosion that is complicated by thrombosis, neutrophil extracellular traps (NETs) are particularly associated, which propagate a low-level inflammation on the luminal endothelium [51]. Specifically, ECs in atherosclerotic plaques express Toll-like receptor 2 (TLR2) which can detect bacterial products as well as extracellular glycosaminoglycans. Atherogenesis involves a disturbed flow, which triggers lesion-specific over-expression of TLR2 in ECs [52]. Exogenous, as well as endogenous ligands such as agonists released during tissue damage or apoptosis, cholesterol crystals or hyaluronic acid, activate TLR2 [51]. Myeloid differentiation primary response gene 88 and other signaling adapters enable TLR-ligation to induce nuclear factor kappa B (NF-κB) activation, which leads to expression of adhesion molecules such as intercellular adhesion molecule 1 (ICAM-1) or E-selectin and chemoattractants, activation of interferon- and proinflammatory cytokine pathways, and production of matrix metalloproteinases [51].

The activation of NF-κB through TLR2 may facilitate disruption of EC-to-extracellular matrix contacts, EC apoptosis, and the desquamation of the endothelial monolayer characteristic of eroded plaque at sites with flow perturbation that favors plaque erosion [51]. Neutrophil recruitment is facilitated by the release of chemokines led by IL-8 from ECs [52]. As neutrophils localize at sites of plaque erosion and then are activated, proteases are released, forming NETs, damaging ECs and trapping leukocytes [51]. It is observed in human specimens that neutrophils are co-localized with patches of TLR2 expression. Consequently, the number of adherent neutrophils is negatively correlated with the continuity of the endothelium, a process that is mediated by TLR2-mediated activation of the ECs [52]. Depletion of neutrophils or inhibition of neutrophil adhesion prevented endothelial erosion, thereby substantiating neutrophils’ role in this pathophysiology.

NETs are composed of unwound nuclear DNA, which is loaded with neutrophil granular proteins, provides a fibrin-like base for platelet adhesion, activation, and aggregation; facilitates the accumulation of prothrombotic molecules, such as vWF and fibrinogen; and contributes to erythrocyte adhesion, all of which potentially lead to thrombus formation [53]. The subendothelial matrix and thrombogenic components may also be exposed when endothelial cells detach, leading to white thrombus formation and vessel occlusion [54].

Besides, ${\mathrm{CD4}}^{+}$ and ${\mathrm{CD8}}^{+}$ T lymphocytes as well as their secretory products such as granulysin, perforin, and granzyme A are localized at the site of plaque erosion compared with plaque rupture [55]. Granzyme A, granulysin, and perforin mediated EC death *ex vivo*, suggesting their involvement in plaque erosion as mediators of direct cytotoxic effects of locally enriched ${\mathrm{CD8}}^{+}$ T lymphocytes [55].

There is another piece to the jigsaw of pathophysiology of plaque erosion provided by an observation which indicates that genetically determined differences may contribute to pro-inflammatory changes in hyaluronan splicing into the pro-inflammatory isoform by a specific enzyme isoform called hyaluronidase 2 (HYAL2), which together with the hyaluronan binding protein (CD44v6) seems to be elevated in plaque erosion, demonstrating a crucial role for hyaluronan in eroded plaques [56].

In summary, there is a growing body of evidence suggesting that, with endothelial activation and dysfunction as the common initiating factor, plaque rupture is lipid-driven and induced by macrophages, whereas endothelial damage is caused by neutrophils, which lead to plaque erosion. Furthermore, an eroded plaque typically forms a thrombus rich in platelets, and more myeloperoxidase-positive inflammatory cells, known as a white thrombus, as opposed to a red thrombus mainly comprising fibrin and erythrocytes that is associated with plaque rupture [57].

In light of the increasing importance of plaque erosion as a hallmark of culprit lesions in ACS, this shift in epidemiology has led to plaque erosion being the primary mechanism in non-ST-segment elevation myocardial infarction (non-STEMI) [58]. As such, plaques with intact fibrous caps, which are considered a separate ACS entity, entail a more tailored treatment approach since plaque erosion exhibits distinct optical, microscopic, and molecular characteristics from plaque rupture.

Although as of today, current standards of care mandate immediate stenting for ST-elevation myocardial infarction, and often an early invasive strategy with stenting for many cases of non-STEMI, which is referred to as such a “one-size-fits-all” clinical strategy [59], the findings from the EROSION study, which investigated the concept of dual antiplatelet treatment with aspirin and ticagrelor without stenting the culprit lesion in patients with residual diameter stenosis <70% on coronary angiography and diagnosed with plaque erosion by optical coherence tomography, and it was found that a 50% decline in thrombus volume was seen in 78.3% of patients after 30 days along with the majority 92.5% of patients remaining free of major cardiovascular events for up to 1 year, provided a proof of concept for the possibility that intensive antithrombotic treatment without stenting may offer patients with plaque erosion a better therapeutic option [60, 61].

In addition, this paradigm change from invasive to noninvasive pharmacological treatment of plaque erosion has been strengthened by recent findings that eroded plaques may even spontaneously heal, resulting in layered appearance of plaque erosion as indicated by optical coherence tomography, but nevertheless require attention with the need for new therapeutic approaches due to their higher local inflammation and vulnerability to developing occlusive thrombosis [62, 63]. Aside from these concepts relevant to antithrombotic treatment, such specific targets as NETosis, HYAL2, myeloperoxidase, and CD44v6 seem promising for plaque erosion therapy [58].

However, even if recent decades have seen significant improvements in the treatment of ASCVD, with early mechanical intervention as well as aggressive lipid modification, unfortunately, there are still some patients who succumb to acute athero-thrombotic events and suffer from residual risks due to poorly controlled inflammation [64, 65].

Recently, the Canakinumab Anti-inflammatory Thrombosis Outcome Study (CANTOS) trial and Low-Dose Colchicine after Myocardial Infarction (COLCOT) trial establish inflammation in atherosclerosis as a clinical reality, showing that lowering the inflammatory burden leads to a reduction in future cardiovascular events irrespective of lipid changes [66, 67, 68]. In CANTOS trial, 10,061 stable patients with previous myocardial infarction and high sensitivity C-reactive protein assay (hsCRP), greater than 2 mg/L, despite maximally tolerated statin treatment were enrolled in the study [66]. Canakinumab, a monoclonal antibody inhibitor of the inflammatory cytokine interleukin (IL)-1β, not only showed significant reductions of IL-6 and hsCRP by up to 40%–60%, but was also linked to markedly reduced major adverse cardiovascular events such as non-fatal myocardial infarction or stroke and cardiovascular death overall by 15%, and by 25% in those with on-treatment hsCRP level lower than 2 mg/L [66, 69]. This was followed by COLCOT trial which investigated a second preventive treatment strategy after recent myocardial infarction using the non-selective anti-inflammatory agent colchicine or placebo. The results of the study showed that a low dose of colchicine of 0.5 mg daily reduced the risk of ischemic ASCVD events (primary endpoint of death, resuscitated cardiac arrest, ACS, stroke, and hospitalization for angina requiring revascularisation) by 23% after a median follow up of 22.6 months compared with placebo in patients recruited in the first 30 days after a myocardial infarction (n = 4755) [67]. The COLCOT observation has recently been extended to patients with stable coronary artery disease in the LoDoCo2 study (n = 5522) [68]. The LoDoCo2 trial, which followed the open-label LoDoCo trial which involved only 532 patients with stable coronary artery disease, demonstrated a similar positive outcome to COLCOT—a reduction of 31% with colchicine 0.5 mg daily in terms of the primary cardiovascular disease endpoint compared to placebo after a median follow-up of 28.6 months [70]. In this respect, low-dose colchicine may now be considered for secondary prevention of cardiovascular disease, particularly if other risk factors are not sufficiently controlled or in the case of recurrent events under optimal therapy according to the 2021 ESC Guidelines on cardiovascular disease prevention in clinical practice [71].

Thus, from being simply a disorder of pathological lipid deposition, our understanding of ASCVD has evolved into a disease that is unarguably triggered by chronic inflammation that initiates a multitude of biochemical and histologic events that result in the initiation and progression of atherosclerotic plaque and the triggering of rupture or erosion causing acute thrombosis [72, 73].

Due to inflammation as a final pathway of risk factors such as hypertension, diabetes, smoking, central obesity, and chronic immune-inflammatory diseases as well as an independent driver of atherogenesis [41, 74], there has been extensive investigation into the link between inflammation and ASCVD, resulting in an ongoing interest which is reflected by the growing number of papers published every year. However, the exponentially increasing volume of publications renders it impossible to identify high-impact research and to keep up to date with the latest findings. In this sense, there is an opportunity to respond to the demands with the bibliometric approaches, since they enable us to explore the structure, productivity, progress, quality, impact, and interconnection of scientific work in greater detail [75, 76].

In this light, the present study aims to identify the major trends in ASCVD and inflammation research with a particular focus on qualitative research at three levels: micro (that of individuals and research groups), meso (the institutional) and macro (the national), the body of knowledge, as well as shifts in research topics with the bibliometric methodology.

## 2. Materials and Methods

### 2.1 Source of the Data and Search Strategy

The search was performed using the Science Citation Index Expanded of the Web of Science Core Collection (WOSCC) of Clarivate Analytics on a single day. The following search strategy was used to identify relevant publications: (((TI=((“heart arrest”) OR (“sudden cardiac death”) OR (“cardiac arrest”) OR (“acute coronary syndrome*”) OR (angina*) OR (coronary NEAR/2 (disease* OR syndrome* OR occlus* OR reocclus* OR re-occlus* OR steno* OR restenos* OR obstruct* OR lesio* OR block* OR harden* OR stiffen* OR obliter* OR thromo*)) OR ((heart OR cardiac OR myocardial) NEAR/2 (isch?em* OR attack* OR infarct*)) OR (“cardiogenic shock”) OR (STEMI OR NonSTEMI OR Non-STEMI OR NSTEMI) OR (“myocardial reperfusion injury”) OR (“arterial occlusive disease*”) OR (arteriosclero* OR arteriolosclero* OR atherosclero*) OR (“peripheral arter* disease*” OR “cerebrovascular accident*”) OR (carotid stenosis) OR (“cerebral vascular disorder*” OR “cerebral vascular disease*” OR “cerebrovasc* disorder*” OR “cerebrovasc* disease*”) OR (((brain* OR cerebral OR lacunar) NEAR/2 (infarct* OR isch?em*)) OR stroke*) OR (brain NEAR/2 accident*) OR apoplexy OR ((intracranial OR intra-cranial) NEAR/2 (hemorrhage* OR emboli* OR thromo*)) OR (ASCVD OR “atherosclerotic CVD” OR “atherosclerotic cardiovascular disease*” OR “atherosclerotic cardio-vascular disease*”) OR (“myocardial revascularization”) OR (angioplast*) OR (“coronary atherectom*”) OR (“coronary artery” NEAR/2 (bypass OR by-pass OR anastomosis)) OR ((cardiac OR heart) NEAR/2 catheterization*) OR (PCI OR “percutaneous coronary intervention*” OR PTCA OR CABG))) AND TS=((inflam* OR “c reactive protein*” OR “acute-phase protein*” OR interleukin* OR “tumo$r necrosis factor*” OR cytokine* OR interferon* OR chemokine*))) AND DT=(Article)) AND LA=(English). The timespan for data retrieval was set from March 10, 2012 until March 10, 2022. The bibliometric information of the publications was collected and imported into CiteSpace and VOSviewer for analysis.

### 2.2 Data Analysis

An information visualization tool, CiteSpace 5.8.R3 (Drexel University, Philadelphia, USA), which was developed by Drexel University Professor Chaomei Chen, was used in the present study. CiteSpace examines three criteria for detecting abrupt changes in the network: burst detection, betweenness centrality, and heterogeneous networks, all of which are able to detect abrupt changes in a timely fashion, indicate a research front’s nature, and label a specialty [77, 78, 79]. VOSviewer v1.6.10.0 (Rapenburg 70, 2311 EZ Leiden, Netherlands), another bibliometric software developed by Professors Van Eck and Waltman from Leiden University, has text mining capabilities to process large-scale data for mapping and clustering of the scientific literature [80].

We evaluated the annual growth of publications with GraphPad Prism 9 software (GraphPad Software Inc., San Diego, CA, USA) in the present study. CiteSpace was used to (a) visualize scientific research cooperation networks involving micro-author cooperation, meso-institutional cooperation, and macro-national cooperation; (b) perform a co-citation analysis of references, and (c) detect the citation bursts of references and keywords. VOSviewer was applied to conduct keyword co-occurrence analysis. The Sankey diagrams (three-field plot) summarized the relationships between prolific authors, their collaborators, and the collaborators’ institutions.

To show acknowledged funders of published research, InCites, available in Web of Science, was used to analyze the resulting publication set.

## 3. Results

### 3.1 Publication Output

**Fig. 1.**
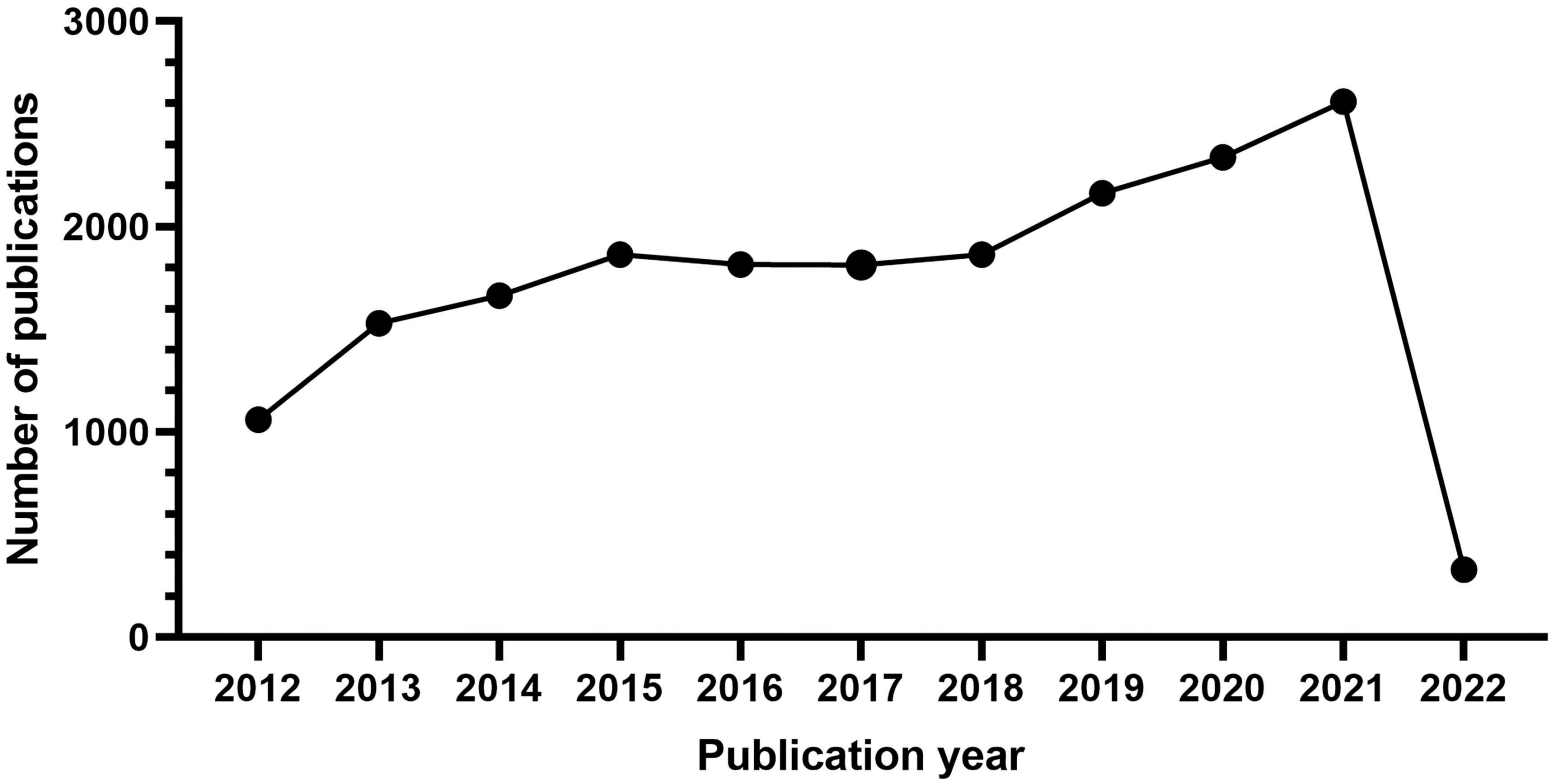
**The number of articles published annually in ASCVD and inflammation research**.

There were 19,053 studies collected for bibliographic records. The number of publications per year is presented in Fig. 1. On the whole, the scientific production from 2012 to 2021 has increased over time and falls into roughly three phases.

From 2012 to 2015, it was the initial period which showed a continuous upward trend, where the number of publications rose from 1061 to 1864. Growth was flat after 2015 and continued until 2018. During the third stage from 2019 through 2021, a period of rapid growth then occurred and the output reached the maximum in 2021 (2609).

### 3.2 Countries or Regions and Institutions Analysis

Between 2012 and 2022, a total of 628 institutions from 135 countries or regions conducted studies related to the field. Table 1 shows the performance of the top 10 countries or regions and institutions. The top 10 countries or regions were primarily distributed across Asia, Europe, and North America; Asia (8654, 45.42%) and Europe (4426, 23.23%) were the top highest-output regions, and North America (4306, 22.60%) followed behind as the third rank. Among them, the top three countries were China (6232, 32.71%), the USA (4306, 22.60%), and Germany (1348, 7.08%).

**Table 1.** **The top 10 countries or regions and institutions involved in ASCVD and inflammation research**.

Rank	Country	Centrality	Count (% of 19,053)	Rank	Institutions	Centrality	Count (% of 19,053)
1	China	0.02	6232 (32.71)	1	Capital Med Univ (China)	0.03	410 (2.15)
2	the USA	0.1	4306 (22.60)	2	Shanghai Jiao Tong Univ (China)	0.01	295 (1.55)
3	Germany	0.01	1348 (7.08)	3	Huazhong Univ Sci & Technol (China)	0.01	279 (1.46)
4	Japan	0.03	1013 (5.32)	4	Shandong Univ (China)	0	270 (1.42)
5	England	0.2	918 (4.82)	5	Harvard Med Sch (the USA)	0	229 (1.20)
6	Italy	0.03	821 (4.31)	6	Univ Washington (the USA)	0.01	228 (1.20)
7	the Netherlands	0.03	746 (3.92)	7	China Med Univ (China)	0.03	227 (1.19)
8	Turkey	0.03	743 (3.90)	7	Nanjing Med Univ (China)	0.03	222 (1.16)
9	South Korea	0	666 (3.49)	8	Karolinska Inst (Sweden)	0.02	220 (1.15)
10	Sweden	0.06	593 (3.11)	9	Harvard Univ (the USA)	0.01	207 (1.09)

As for the analysis of institutions, the leading research organization for publications on this topic was Capital Med Univ (410, 2.15%), followed by Shanghai Jiao Tong Univ (295, 1.55%), and Huazhong Univ Sci & Technol (279, 1.46%).

**Fig. 2.**
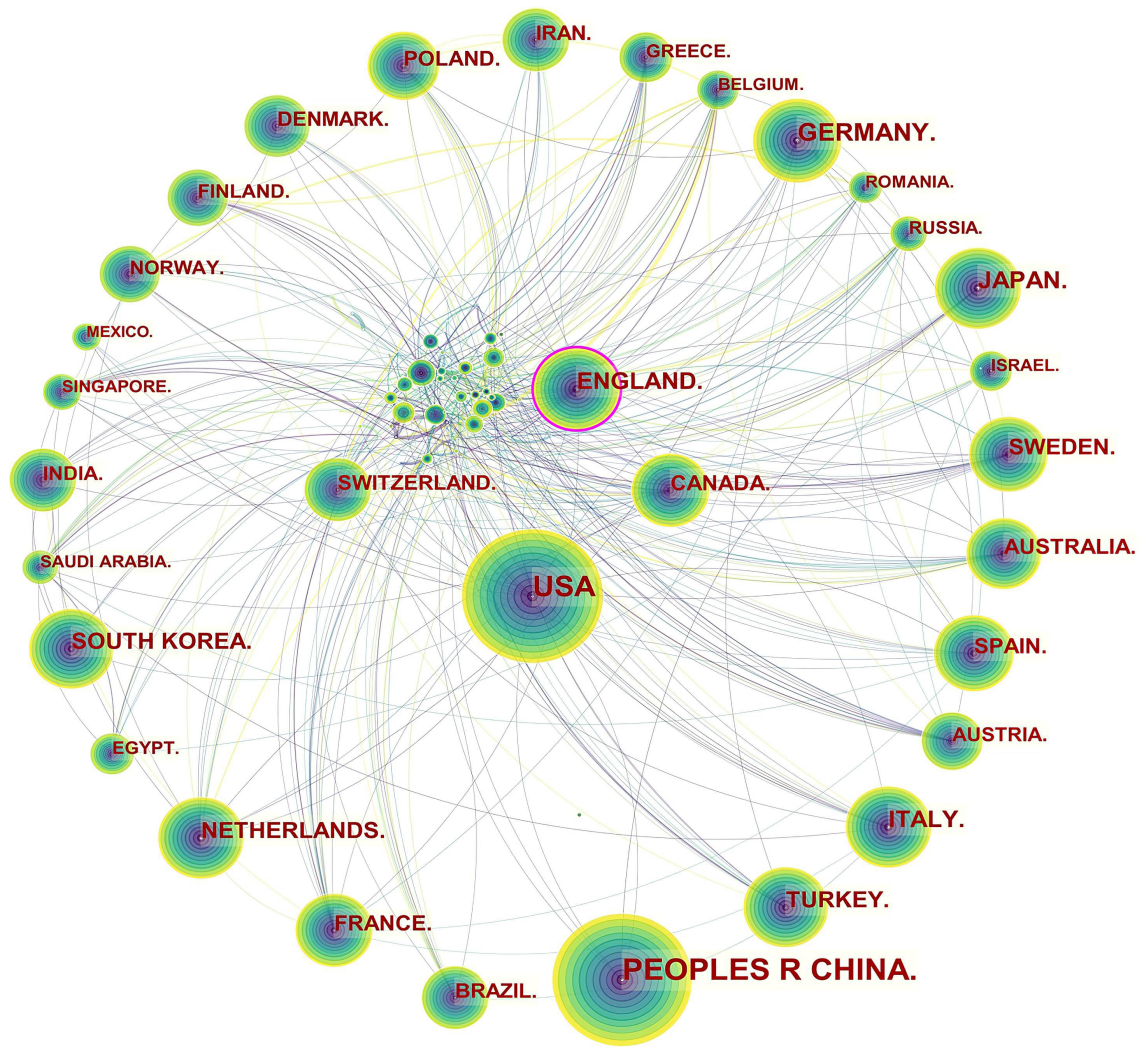
**Network of countries and regions engaged in ASCVD and inflammation research**.

Specifically, three levels of scientific collaboration network analysis are presented, namely the micro-author, the meso-institutional, and the macro-national. In Fig. 2, each node represents a country or region, and the volume associated with each node corresponds to the number of publications it shares. There is a bidirectional relationship between the two countries as indicated by the connecting curve, and the thickness of the curve signifies the strength of the bidirectional cooperation. The nodes with a high betweenness centrality greater than 0.1 (e.g., those linked with over 10% of the nodes in the entire network) are identified by purple rings. The more connections an individual nation possesses, the greater its influence or betweenness centrality is in the network [81]. For example, the USA cooperated frequently with Canada, Mexico, Argentina, Colombia, Peru, Spain, South Korea, China, Thailand, Japan, Iran, Lebanon, Jordan, Australia, Ethiopia, and Uganda. England worked closely with the USA, Canada, Uruguay, Finland, Norway, Italy, Luxembourg, Hungary, Sweden, France, Denmark, Germany, Greece, the Netherlands, Czechia, Sweden, Switzerland, Romania, Serbia, Spain, Malaysia, Singapore, Iran, India, Pakistan, Malawi, South Africa, Nigeria, and Ghana.

The publication volumes of Canada (n = 537) and Switzerland (n = 314) did not place them among the top ten, ranking as the twelfth and nineteenth, respectively, but they maintained extensive collaborative relationships in this field. The main collaborators with Canada were the USA, Cuba, Mexico, Ecuador, Luxembourg, Switzerland, Iceland, Poland, France, Switzerland, Sri Lanka, Iran, Philippines, Lebanon, Oman, Singapore, Israel, Saudi Arabia, United Arab Emirates, Thailand, Vietnam, Kuwait, and Australia. The main countries that collaborated with Switzerland were Canada, Brazil, the Netherlands, Czechia, England, Hungary, Belgium, Bulgaria, Luxembourg, Liechtenstein, Italy, Spain, Germany, France, Latvia, Denmark, Russia, Senegal, South Africa, Israel, Turkey, and Japan.

**Fig. 3.**
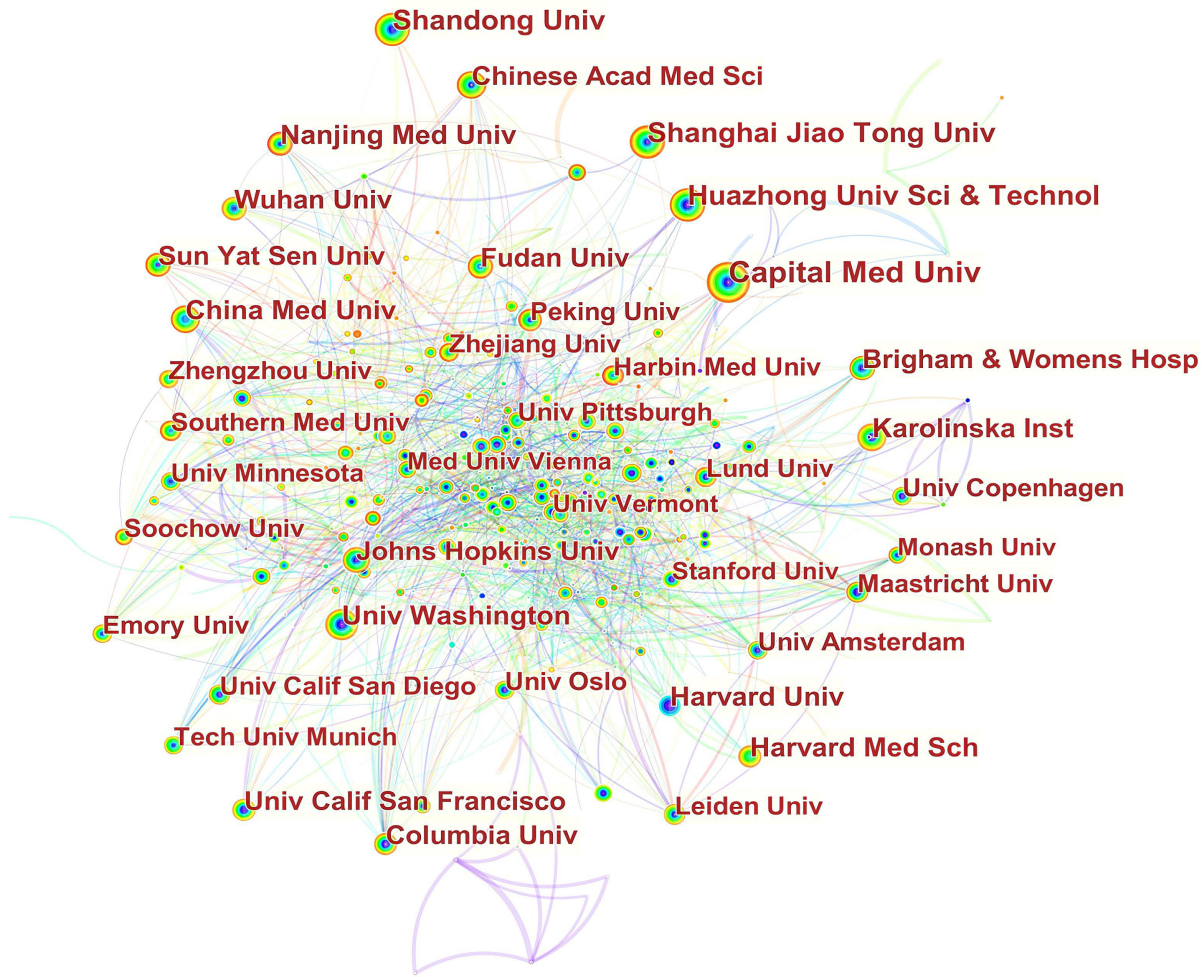
**Network of institutions engaged in ASCVD and inflammation research**.

In Fig. 3, it can be seen that inter-institutional collaborations have remained globally scattered, with cooperation between domestic institutions being closer. For example, Capital Med Univ had close cooperation with China Acad Chinese Med Sci,China Med Univ, China Acad Chinese Med Sci, China Natl Clin Res Ctr Neurol Dis, Beijing Inst Heart Lung & Blood Vessel Dis, Beijing Inst Brain Disorders, Peoples Hosp Guangxi Zhuang Autonomous Reg, Chinese Acad Med Sci & Peking Union Med Coll. Jilin Univ, and Peking Univ. China Med Univ cooperated frequently with Capital Med Univ, Chang Gung Univ, Chung Shan Med Univ, China Med Univ Hosp, Taipei Med Univ Hosp, I Shou Univ, Zhejiang Univ, and Asia Univ.

### 3.3 Authors

A total of 594 authors published papers related to ASCVD and inflammation. As shown in Table 2, Christian Weber was the top contributor (75, 0.39%), followed by Mary Cushman (46, 0.24%), and Edward A Fisher (39, 0.20%). The top authors by betweenness centrality were Christie M Ballantyne (0.14) and Peter Libby (0.12).

**Table 2.** **The top 10 authors of ASCVD and inflammation research**.

Rank	Author	Count (% of 19,053)	Centrality
1	Christian Weber (Germany)	75 (0.39)	0.04
2	Mary Cushman (the USA)	46 (0.24)	0.01
3	Edward A Fisher (the USA)	39 (0.20)	0.07
4	Pål Aukrust (Norway)	38 (0.20)	0.01
4	Esther Lutgens (Germany)	38 (0.20)	0.05
5	Christie M Ballantyne (the USA)	36 (0.19)	0.14
6	François Mach (Switzerland)	31 (0.16)	0.01
6	Peter Libby (the USA)	31 (0.16)	0.12
7	Myung Ho Jeong (South Korea)	30 (0.16)	0
7	Jan Nilsson (Sweden)	30 (0.16)	0.01
7	Matthias Nahrendorf (the USA)	30 (0.16)	0.06
8	Gerard Pasterkamp (the Netherlands)	29 (0.15)	0.01
9	Michael J Blaha (the USA)	28 (0.15)	0.01
9	Yun Zhang (China)	28 (0.15)	0.02
9	Ziad Mallat (England)	28 (0.15)	0.06
10	Aaron R Folsom (the USA)	26 (0.14)	0.01

**Fig. 4.**
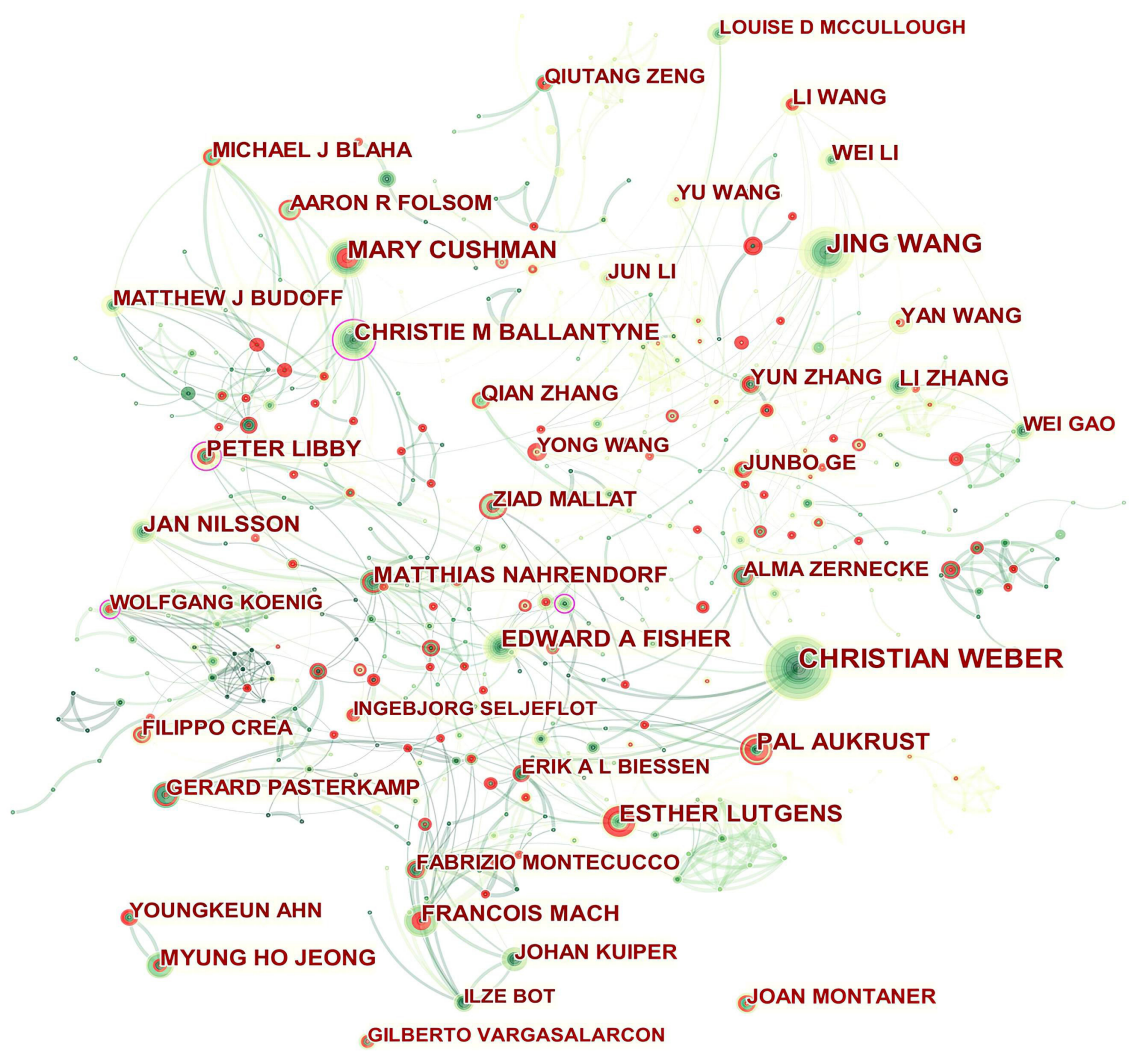
**Network of authors in ASCVD and inflammation research**.

Fig. 4 presents the authors’ collaboration network. Isolated authors or authors with no connecting curves with others are devoid of any collaboration. Christie M Ballantyne, Peter Libby, Wolfgang Koenig, and Oliver Soehnlein played a central role in their respective collaborating networks. We thus took a closer look at their collaborative community as shown in **Supplementary Figs. 1**–**4** by Sankey diagrams. The Sankey diagrams list these prolific authors (left field), their collaborators (middle field), and their co-authors’ institutions (right field). The Sankey diagram is a type of flowchart in which the width of the band shows the amount of flow, so the wider the band, the greater the flow quantity. As a result, these figures also indicate the number of publications that have been co-authored.

In addition, Qiutang Zeng, Junbo Ge, Yun Zhang, Mary Cushman, Ingebjørg Seljeflot, Filippo Crea, Pål Aukrust, Esther Lutgens, François Mach, Youngkeun Ahn, and Joan Montaner who were detected with strong bursts revealed high scholarly activity recently.

### 3.4 Journals and Co-Cited Academic Journals

The field of ASCVD and inflammation was covered by 1979 journals. The top 10 productive journal outlets which pooled 3095 (16.24%) papers are presented in Table 3.

**Table 3.** **Top 10 journal and top 10 co-cited journals in ASCVD and inflammation research**.

Rank	Journal	Count (% of 19053)	IF	JCR	Rank	Co-cited Journal	Count (% of 471016)	IF	JCR
1	PloS one (the USA)	706 (3.71)	3.752	Q2	1	Circulation (the USA)	13276 (2.81)	39.918	Q1
2	Atherosclerosis (Ireland)	492 (2.58)	6.847	Q1	2	Arteriosclerosis, thrombosis, and vascular biology (the USA)	8065 (1.71)	10.514	Q1
3	Scientific reports (England)	347 (1.82)	4.996	Q2	3	The New England journal of medicine (the USA)	7983 (1.70)	176.079	Q1
4	Arteriosclerosis, thrombosis, and vascular biology (the USA)	345 (1.81)	10.514	Q1	4	Journal of the American College of Cardiology (the USA)	7931 (1.68)	27.203	Q1
5	Journal of the American Heart Association (England)	254 (1.33)	6.106	Q2	5	Atherosclerosis (Ireland)	7741 (1.63)	6.847	Q1
6	International journal of cardiology (the Netherlands)	238 (1.25)	4.039	Q2	6	PloS one (the USA)	7396 (1.57)	3.752	Q2
7	Journal of stroke and cerebrovascular diseases: the official journal of National Stroke Association (the USA)	197 (1.03)	2.677	Q3	7	Circulation research (the USA)	6847 (1.45)	23.213	Q1
8	Stroke (the USA)	191 (1.00)	10.17	Q1	8	European heart journal (England)	5884 (1.25)	35.855	Q1
9	Experimental and therapeutic medicine (Greece)	173 (0.91)	2.751	Q4	9	Stroke (the USA)	5474 (1.16)	10.17	Q1
10	Circulation research (the USA)	152 (0.79)	23.213	Q1	10	Lancet (England)	5422 (1.15)	202.731	Q1

The top three prolific journals were *PloS one* (706, 3.71%), *Atherosclerosis* (492, 2.58%) and *Scientific reports* (347, 1.82%). Publishers of the productive journals are located in the USA and Europe (Ireland, England, the Netherlands, and Greece). These journals had an impact factor (IF) ranging from 2.677 to 23.213. Moreover, 80% of the active journals were classified as Q1 or Q2.

Referencing other scientific publications is a regular feature of scientific publications. This generates further networks, such as bibliographic coupling or co-citation networks. It is through these networks that meaningful properties of the underlying research system are captured, and specifically, the influence of different bibliometric units, such as documents and journals, are determined. Bibliographic coupling occurs when at least one cited source appears in both articles’ bibliographies or reference lists [82]. The co-citation of two articles occurs when both are cited in a third article [83, 84]. As such, co-citation serves as a counterpart to bibliographic coupling.

Co-citation analysis is an established method for identifying research domains, for example the research orientations in a field, and it is based on the assumption that the references cited together in an article have intellectual affinities [83, 84]. A co-citation can be examined at different levels: the publications per se, the cited authors, and the cited journals. A journal co-citation analysis calculates the frequency with which articles from two journals are co-cited in other articles. The high co-citation of two journals indicates that the two journals have a strong semantic relationship; in addition, high co-citations of a journal are indicative of this journal being a prominent source containing papers concerning ASCVD and inflammation, which have been co-cited by other articles.

Similar to productive journals, the top 10 co-cited journals were from the USA and Europe (Ireland, England, the Netherlands, and Greece). The highest-ranking journal was *Circulation*, with 13,276 co-citations and an IF of 39.918. The second was *Arteriosclerosis, thrombosis, and vascular biology*, with 8065 co-citations and an IF of 10.514. The third was *The New England journal of medicine*, with 7983 co-citations and an IF of 176.079. All the highly co-cited journals had an IF above 5.000 excluding *PloS one* (3.752 IF).* Lancet* had the highest IF of 202.731. In addition, journals in the Q1 accounted for 90% of the highly co-cited journals. There is a concurrence of *PloS one*, *Atherosclerosis*, *Arteriosclerosis, thrombosis, and vascular biology*,* Stroke*, and *Circulation research *in the prolific journals and highly co-cited ones.

### 3.5 Acknowledged Funders

Our next step was to identify which organizations are acknowledged funders of published research. InCites offers analysis opportunities for identifying and analyzing the funders of published research based on their acknowledgments. However, not all publications listed their funding sources: 10570 of the 19053 publications in our dataset acknowledged a funder, which equals 55.48%. In addition, Web of Science may not have collected the funding data for some of the older publications included in this set, explaining the absence of acknowledged funders.

**Fig. 5.**
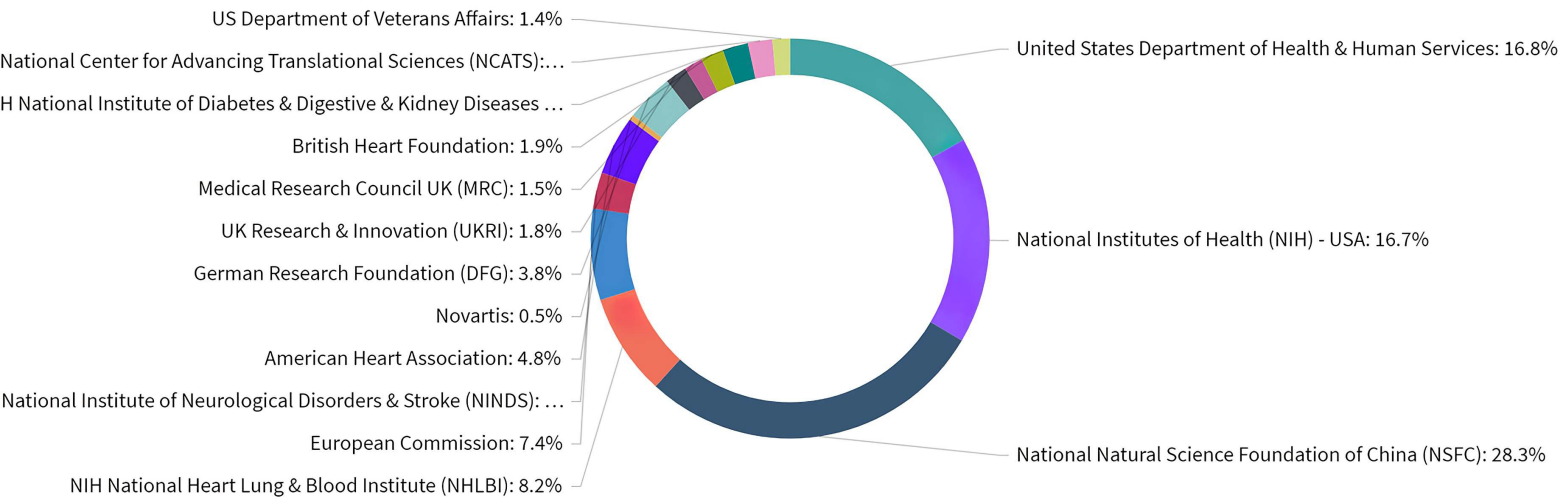
**Distribution of publications over all acknowledged research funders in ASCVD and inflammation research**.

Overall, ASCVD and inflammation research was funded by a variety of organizations as shown in Fig. 5. Based on the publications that acknowledged a funder, 28.3% of publications have been funded by the National Natural Science Foundation of China (NSFC). In addition, NIH consists of 27 individual institutes and centers, each with their own research agenda, focusing on a different area of research. In regards to ASCVD and inflammation research in the USA, various institutes and centers are recognized as funders, with research emphasis ranging from cardiology, respirology, and haematology to nephrology, diabetology, and gastroenterology, reflecting the variety of lines of research. European funding sources from Great Britain, Germany, and Switzerland were also active in this field.

**Fig. 6.**
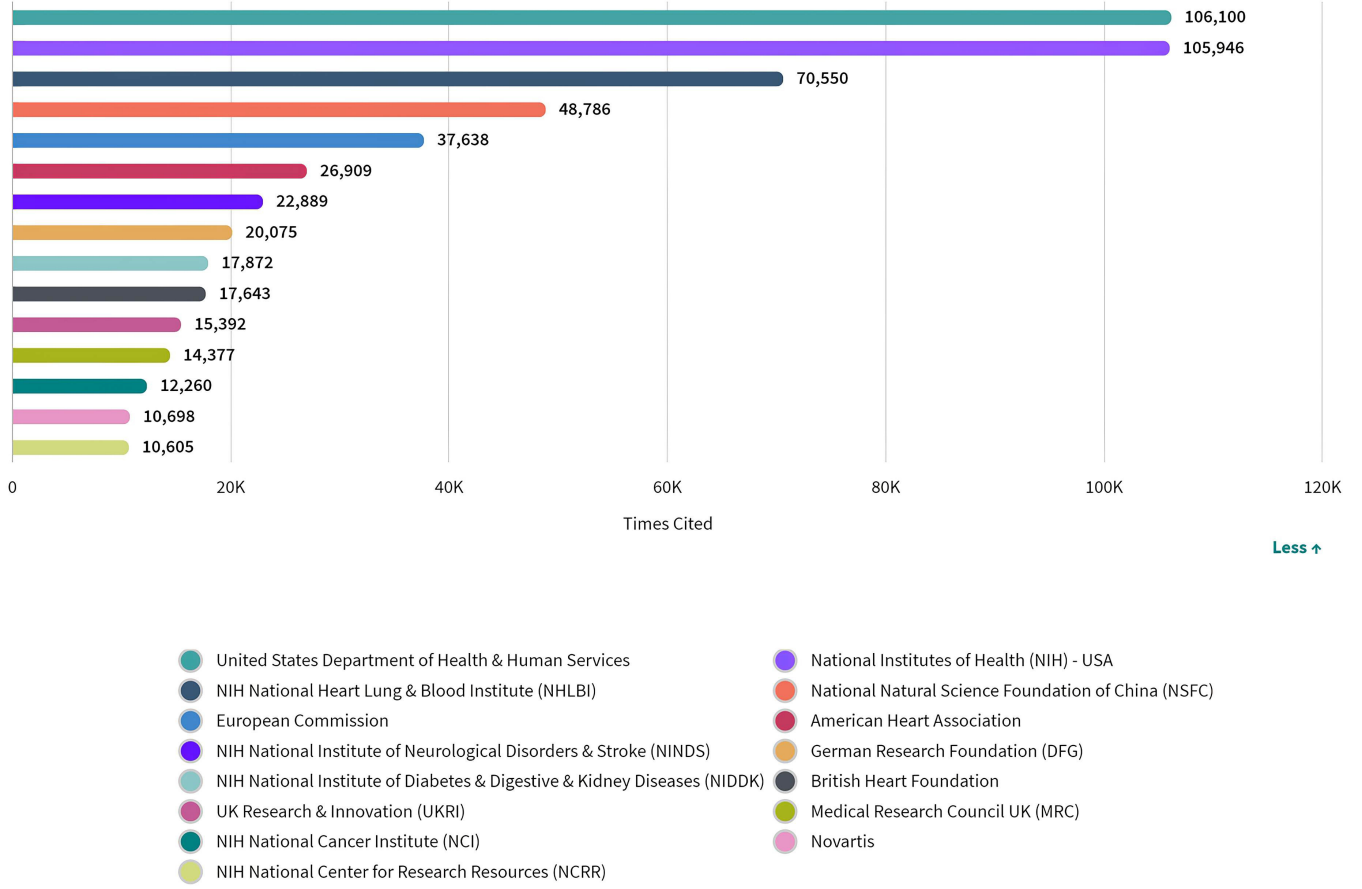
**Number of citations of publications by funding agencies in ASCVD and inflammation research**.

Although citations cannot provide an explanation for why researchers cited a particular paper, nor can they reflect the quality of research and the conclusions described in a manuscript, they do provide an indication of the reputation of an entity as a contributor to the field [85]. An author’s or group’s credit and the impact of a particular work can be proportionately related to the number of citation records, that is, a higher number of citation records is an indication of the manuscript’s contribution to the current body of knowledge [86]. Therefore, we looked at the the number of citations in this field by funding sources (Fig. 6). According to the analysis, there was no identifiable correlation between the number of citations and the number of publications funded by a single source, since the number of citations received by articles supported by funding bodies of the USA ranked first, despite the fact that the USA came second in terms of publication volume.

### 3.6 Co-Cited References and References with Citation Bursts

In Table 4, we presented the most co-cited papers among the 1185 co-cited references. Of these references, *Antiinflammatory Therapy with Canakinumab for Atherosclerotic Disease * [66], published in *The New England journal of medicine*, was the most co-cited (555, 2017), followed by *Efficacy and Safety of Low-Dose Colchicine after Myocardial Infarction* [67], published in *The New England journal of medicine* (123, 2019), and *Local proliferation dominates lesional macrophage accumulation in atherosclerosis* [87], published in *Nature medicine* (105, 2013).

**Table 4.** **Top 10 co-cited references in ASCVD and inflammation research**.

Rank	Reference	Journal	Citation	Year
1	Antiinflammatory Therapy with Canakinumab for Atherosclerotic Disease	*The New England journal of medicine*	555	2017
2	Efficacy and Safety of Low-Dose Colchicine after Myocardial Infarction	*The New England journal of medicine*	123	2019
3	Local proliferation dominates lesional macrophage accumulation in atherosclerosis	*Nature medicine*	105	2013
4	Myocardial infarction accelerates atherosclerosis	*Nature*	89	2012
5	Low-Dose Methotrexate for the Prevention of Atherosclerotic Events	*The New England journal of medicine*	72	2019
6	Evolocumab and Clinical Outcomes in Patients with Cardiovascular Disease	*The New England journal of medicine*	71	2017
7	Relationship of C-reactive protein reduction to cardiovascular event reduction following treatment with canakinumab: a secondary analysis from the CANTOS randomised controlled trial	*Lancet*	67	2018
8	Large-scale association analysis identifies new risk loci for coronary artery disease	*Nature genetics*	64	2013
9	A prospective natural-history study of coronary atherosclerosis	*The New England journal of medicine*	63	2011
10	NLRP3 inflammasomes are required for atherogenesis and activated by cholesterol crystals	*Nature*	62	2010

As shown in Table 5, the highest-ranked co-cited references by betweenness centrality were published from 2010 to 2015. Among them, *Local proliferation dominates lesional macrophage accumulation in atherosclerosis* [87], published in *Nature medicine*, received the highest betweenness centrality (0.35), followed by *Myocardial infarction accelerates atherosclerosis * [88], published in *Nature* (0.24), and *Detecting human coronary inflammation by imaging perivascular fat* [89], published in *Science translational medicine *(0.22).

**Table 5.** **Top 5 co-cited references with the highest betweenness centrality in ASCVD and inflammation research**.

Rank	Reference	Journal	Centrality	Year
1	Local proliferation dominates lesional macrophage accumulation in atherosclerosis	*Nature medicine*	0.35	2013
2	Myocardial infarction accelerates atherosclerosis	*Nature*	0.24	2012
3	Detecting human coronary inflammation by imaging perivascular fat	*Science translational medicine*	0.22	2017
4	Identification of a novel macrophage phenotype that develops in response to atherogenic phospholipids via Nrf2	*Circulation research*	0.2	2010
4	Brown fat activation reduces hypercholesterolaemia and protects from atherosclerosis development	*Nature communications*	0.2	2015
5	Microglia/macrophage polarization dynamics reveal novel mechanism of injury expansion after focal cerebral ischemia	*Stroke*	0.18	2012

**Fig. 7.**
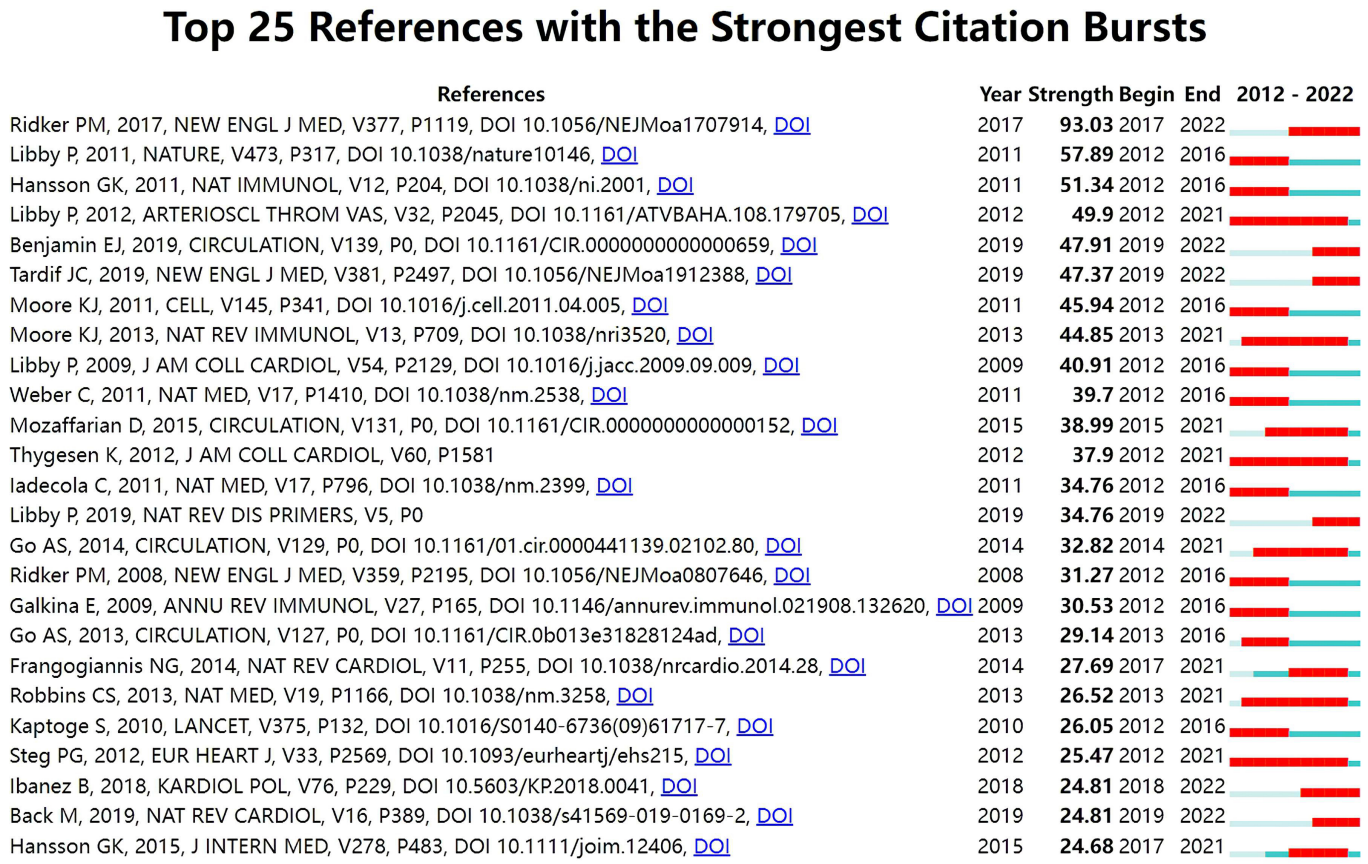
**Top 25 references with strong citation bursts in ASCVD and inflammation research**.

With Kleinberg’s algorithm, burst detection is able to model the times and strengths when certain features gain a lot of prominence. The top 25 references with the strongest citation bursts are shown in Fig. 7. In the map, Year represents the earliest year in which the reference appeared. Strength represents the citation strength. Time interval is shown as a blue line. The red segment shows the year of the beginning and end of each citation burst. The reference with the strongest citation burst of 93.03 entitled “*Antiinflammatory Therapy with Canakinumab for Atherosclerotic Disease*” published in *The New England journal of medicine* was written by Ridker PM *et al*. [66], followed by *Progress and challenges in translating the biology of atherosclerosis * [90], published in *Nature*, with a citation burst of 57.89, and *The immune system in atherosclerosis * [91], published in *Nature immunology*, with a citation burst of 51.34.

### 3.6 Keywords Analysis

A keyword refers to a term that captures the essence of the topic of a document. Here we studied the keywords, considering them essential indicators of the underlying concepts in ASCVD and inflammation research. In total, 1152 keywords were extracted after excluding irrelevant keywords and combining keywords that had the same semantic meaning.

Table 6 presents the meaningful keywords that most frequently occurred in ASCVD and inflammation research. These keywords included low grade inflammation (5643, 0.01), cardiovascular risk factor (3865, 0), gene expression (3293, 0.01), coronary artery disease (3068, 0), CRP (2098, 0), acute myocardial infarction (1964, 0), and mortality (1404, 0).

**Table 6.** **Top 20 keywords with the highest count in ASCVD and inflammation research**.

Rank	Keywords	Count	Centrality	Rank	Keywords	Count	Centrality
1	low grade inflammation	5643	0.01	11	endothelial dysfunction	1006	0
2	cardiovascular risk factor	3865	0	12	oxidative stress	967	0.01
3	gene expression	3293	0.01	13	macrophage activation	852	0
4	coronary artery disease	3068	0	14	chronic heart failure	843	0
5	atherogenesis	2348	0	15	LDL	793	0
6	CRP	2098	0	16	TNF-α	772	0
7	acute myocardial infarction	1964	0	17	ischemia reperfusion injury	759	0.01
8	mortality	1404	0	18	NF-κB	695	0
9	focal cerebral ischemia	1385	0		apoptosis	654	0
10	acute ischemic stroke	1294	0	19	VSMC	639	0

The co-occurrence concept is described as an indicator of a certain similarity and a close relation between the used keywords when the keywords co-occur in documents [92]. A keyword co-occurrence analysis not only identifies the most common keywords used by authors but can also help uncover research trends and explore the research landscape [93, 94, 95].

**Fig. 8.**
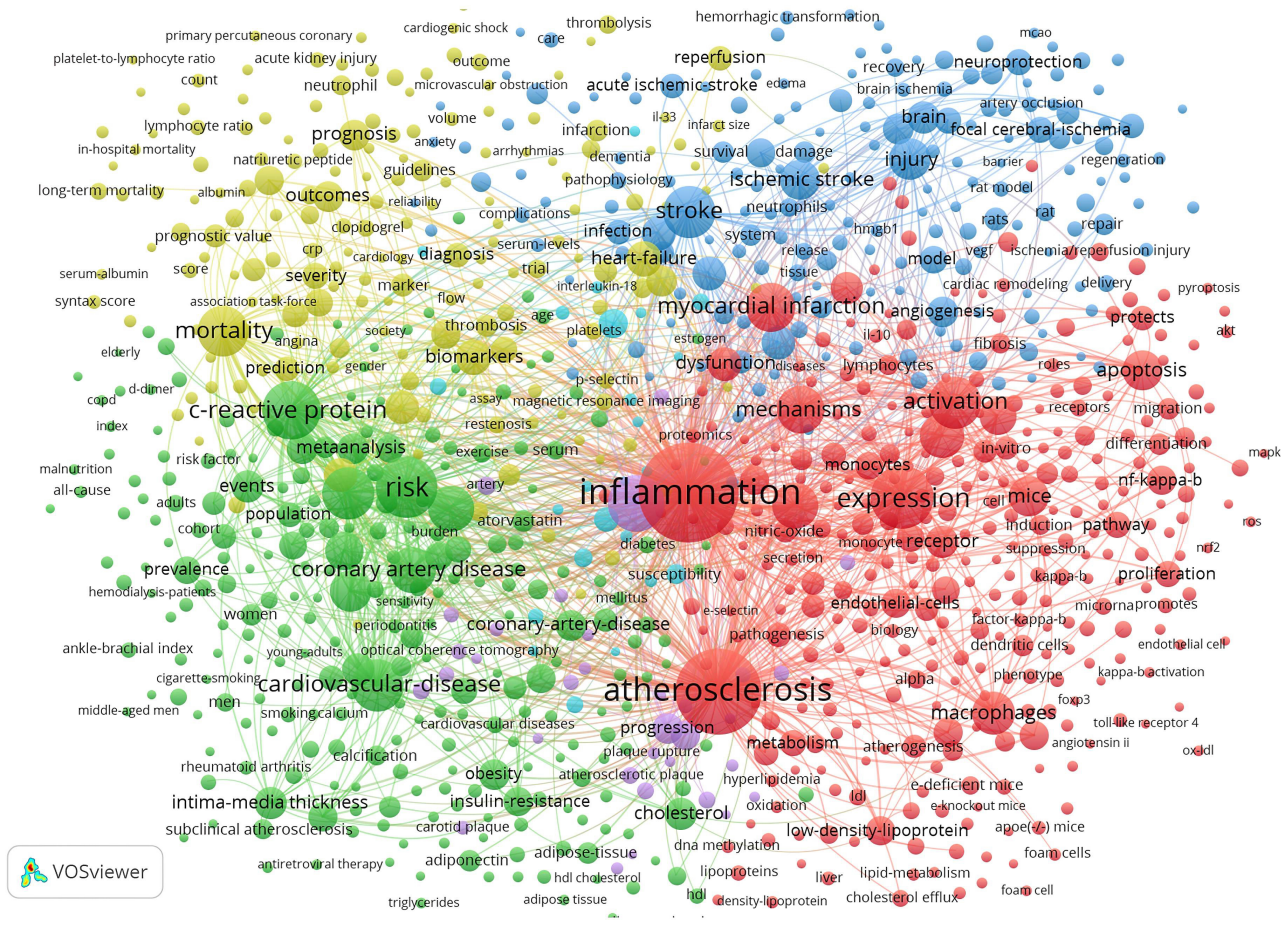
**Map of keyword clustering with a minimum of 5 co-occurrences in ASCVD and inflammation research**.

Fig. 8 depicts the keyword co-occurrence network graph generated by VOSviewer. A keyword is represented by a node. A node’s size indicates the number of occurrences, and the thickness of a line indicates the frequency at which two keywords are linked. Keywords sharing the same colors are grouped into semantically related themes. These are divided into four clusters: the molecular mechanism behind inflammation during the ischemic stroke (blue cluster); inflammatory processes and cellular participants in atherosclerosis (red cluster); markers for the diagnosis and prognosis in ASCVD (yellow cluster); and clinical scenarios related to ASCVD (green cluster).

**Fig. 9.**
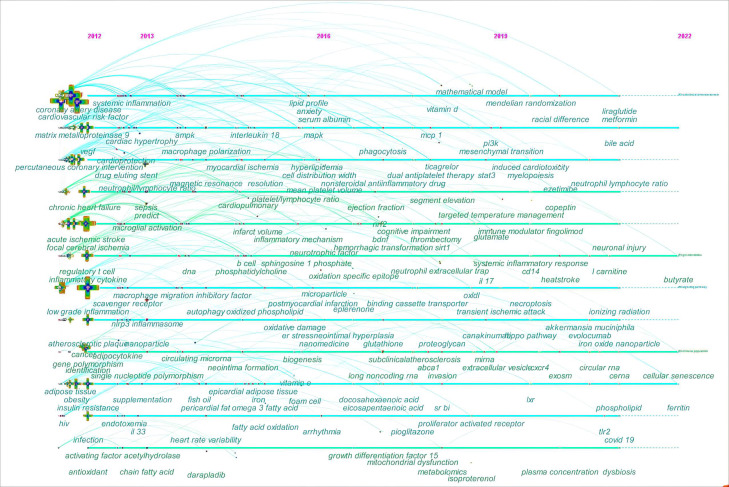
**The timeline view of keyword co-occurrence in ASCVD and inflammation research**.

In the fields of bibliometrics, through analysis of co-occurring keywords, the evolutionary trends can be quickly grasped for a specific research field. Thus, the keyword co-occurrence was further analyzed on a timeline view in Fig. 9. The timeline view consists of keywords on the vertical axis and the years on the horizontal axis. Each keyword is represented by a node. A series of lines are used to highlight the years when keywords co-occur.

In the early years from 2012 to 2016, this field began to focus on (1) sepsis; (2) anxiety; (3) fingolimod and darapladib; (4) nanomedicine; (5) vitamin E and lipoprotein(a); (6) long-chain omega-3 polyunsaturated fatty acids, phosphatidylcholine, sphingosine-1-phosphate, and oxidized phospholipid; (7) epicardial adipose tissue; (8) neutrophil-lymphocyte ratio, platelet-to-lymphocyte ratio, and mean platelet volume; (9) insulin resistance; (10) intestinal microbiota, short-chain fatty acid (SCFA), and lipopolysaccharides (LPS); (11) microparticle; (12) human immunodeficiency virus; (13) neointima formation; (14) Treg cells and B cell; (15) antioxidant; (16) autophagy, oxidative stress, and endoplasmic reticulum stress; (17) microglial activation and macrophage polarization; (18) single nucleotide polymorphism and DNA methylation; (19) matrix metalloproteinase 9; (20) IL-18; (21) vascular cell adhesion molecule-1 (VCAM-1) and intercellular adhesion molecule-1; (22) vascular endothelial growth factor; (23) nucleotide-binding oligomerization (NOD), Leucine-rich repeat (LRR)-containing protein (NLRP3) inflammasome; (24) mitogen-activated protein kinase (MAPK) and NF-κB.

In the mid-term phase, from 2016 to 2019, researchers began to focus efforts on (1) metabolomics; (2) pioglitazone, dipeptidyl peptidase-4 inhibitor, canakinumab, ticagrelor, and eplerenone; (3) vitamin D; (4) decosahexaenoic acid and eicosapentaenioc acid; (5) microvascular endothelial cell; (6) glutathione; (7) trimethylamine N-oxide (TMAO); (8) proteoglycan; (9) clonal hematopoiesis; (10) neointimal hyperplasia; (11) epithelial to mesenchymal transition and mitochondrial dysfunction; (12) NETs, extracellular vesicles (EVs), and exosomes; (13) IL-17, monocyte chemoattractant protein-1, and growth differentiation factor-15; (14) scavenger receptor class B type I and peroxisome proliferator-activated recptor-γ; (15) brain-derived neurotrophic factor and ATP-binding cassette transporter A1; (16) microRNA; (17) sirtuin-1, B cell lymphoma-2, signal transducer and activator of transcription 3, phosphoinositide 3-kinase, and nuclear factor erythroid 2-related factor 2 (Nrf-2).

From 2019 to 2022, the field turned to research on (1) mendelian randomization; (2) COVID-19; (3) iron oxide nanoparticle; (4) liraglutide and metformin; (5) traditional Chinese medicine; (6) canakinumab, ezetimibe, and evolocumab; (7) ionizing radiation; (8) neutrophil-lymphocyte ratio; (9) perivascular adipose tissue; (10) *Akkermansia muciniphila*; (11) L-carnitine, bile acid, choline, and butyrate; (12) ferritin and copeptin; (13) glutamate; (14) myelopoiesis; (15) dysbiosis and endotoxin; (16) necroptosis, cellular senescence, efferocytosis, and microglia/macrophage polarization; (17) TLR2, CD14, and liver X receptor; (18) long non-coding RNAs (lncRNAs), circular RNAs, and competitive endogenous RNAs; (19) microvesicles; (20) proprotein convertase subtilisin/kexin type; (21) Hippo signaling pathway, extracellular signal-regulated kinase, gasdermin D, and caspases; (22) hypoxia-inducible factor-1 and chemokine C-X-C motif ligand 12.

**Fig. 10.**
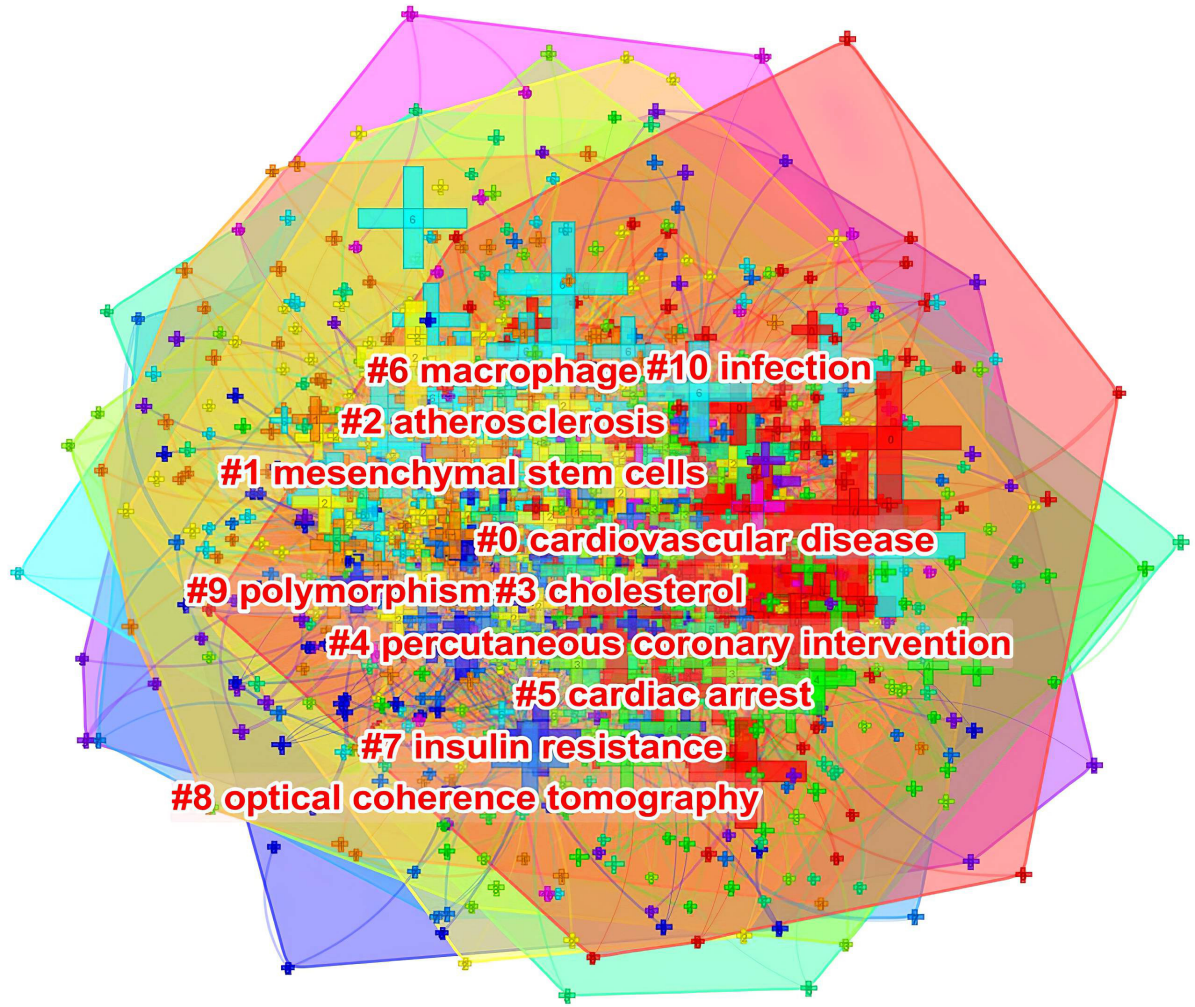
**The keyword clustering knowledge map of ASCVD and inflammation research**.

As shown in Fig. 10, keywords shown in the same color are closely related and clustered together. The cluster was assigned a tag #, with a decreasing number representing more keywords included in the cluster. The cluster label represents the key areas of research. The following 10 clusters were presented: #0 cardiovascular disease; #1 mesenchymal stem cells; #2 atherosclerosis; #3 cholesterol; #4 percutaneous coronary intervention; #5 cardiac arrest; #6 macrophage; #7 insulin resistance; #8 optical coherence tomography; #9 polymorphism; and #10 infection.

**Fig. 11.**
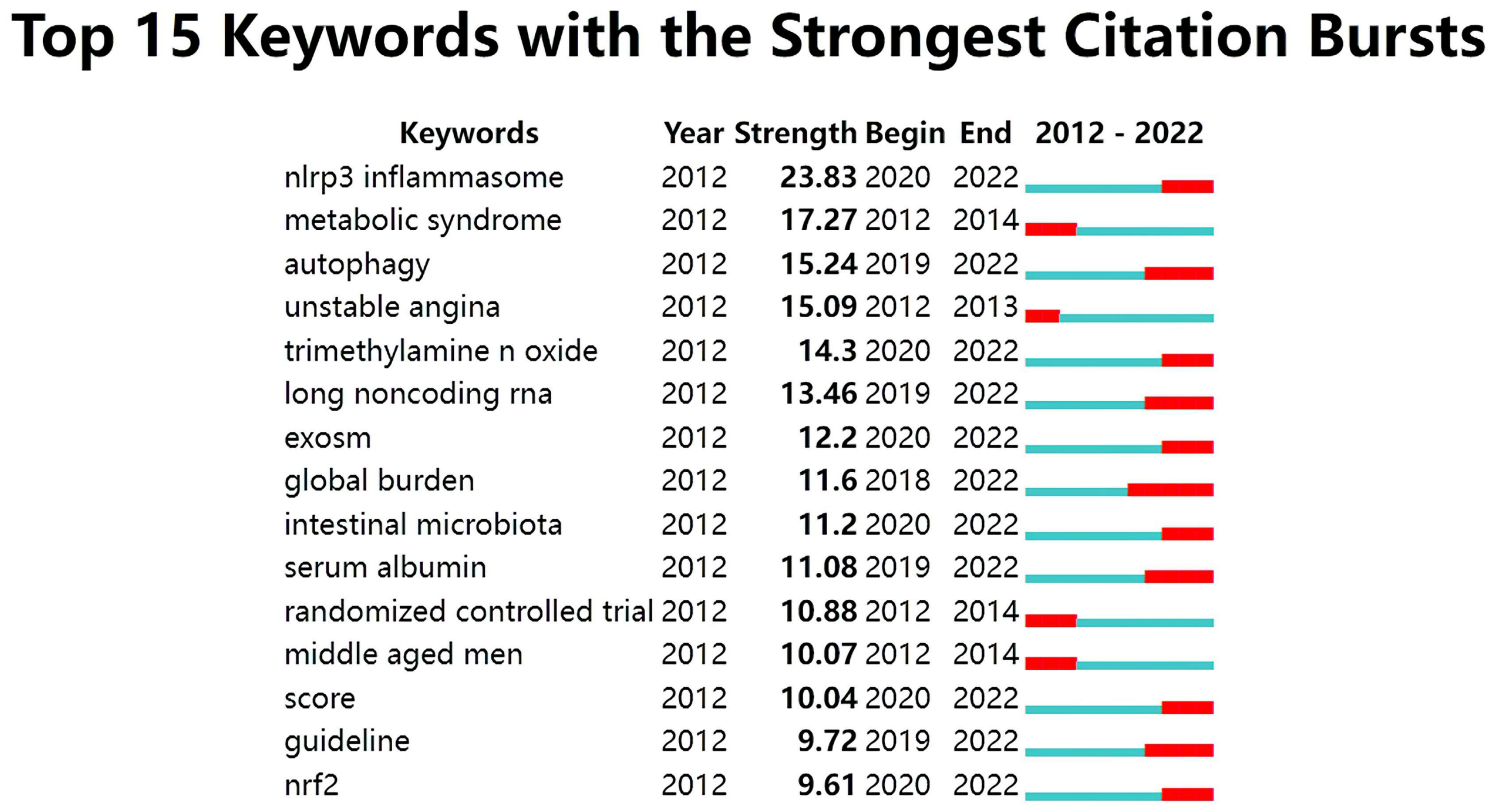
**Top 15 keywords with strong citation bursts in ASCVD and inflammation research**.

The analysis of citation bursts was used to identify keywords that experienced a surge of appearances or citations during a defined time period. As shown in Fig. 11, the results revealed that the top keywords ranked by the strength of citation bursts were “NLRP3 inflammasome” (23.83), “metabolic syndrome” (17.27), “autophagy” (15.24), and “unstable angina” (15.09), etc.

## 4. Discussion

### 4.1 General Information

In Fig. 1, the observed annual growth rate of publications revealed the continuous attention this field has gained annually at a global level. Notably, an outbreak in ASCVD and inflammation research was witnessed during the third stage from 2019 through 2021. The decline in 2022 may reflect a time-lag between publications and indexing in WoS database and year 2022 was still open for new issues [96]. A significant impact of the COVID-19 pandemic in this field can also be inferred; in particular, basic scientific research has been hit particularly hard because of the nationwide lockdown amid the COVID-19 epidemic [97]. Moreover, a decline in opportunities to conduct research is also anticipated due to a long-term economic downturn caused by COVID-19’s looming lockdown, making research funding a source of concern even further due to the reduction in funds available for research [98]. Nevertheless, it seems possible that the field is about to enter its golden period and there are interesting findings that should be noted in recent years.

Table 1 indicates that Asian and European countries were the main providers of publications in the field. A node with a high betweenness centrality (more than 0.1) tends to exert a great deal of influence over the flow of items through the network [99]. Based on the high betweenness centrality, it is evident that some countries dominated in the field (e.g., the USA and England). Although Asian countries stood out as research producers, the betweenness centrality was close or equal to zero, therefore, indicating that China, Japan, South Korea, and Turkey wielded a lower influence.

The vast majority of active institutions are located in Asia and North America. However, research capacity at the top productive institutions was generally weak since none of them exhibited a high betweenness centrality exceeding 0.1; they were thus not considered as major entities controlling significant resources in their collaborative networks, and their publications were less likely to have influenced other research in this decade [100].

The notion of scientific cooperation is described by Katz and Martin as an interdisciplinary collaboration of scholars with the goal of generating new scientific knowledge [101]. By collaborating with more authors, researchers are able to produce more influential papers [102]. Fig. 2 indicated a good level of collaborative work, forming a world map of research collaborations that was centered in North American and European countries. Instead, Asian countries with high scientific output had fewer international collaborations, which tended to be intra-continental phenomena. For example, China, as the leading publication provider, collaborated with Japan, Qatar, Nepal and the USA, whose collaborations lacked diversification. It is possible that scientific advances in ASCVD and inflammation research in Asian countries were plagued by the poor performance of transnational cooperation and academic exchange.

In Fig. 3, in terms of international collaboration, data found there was a weak degree of international collaboration between institutions. There is a tendency for institutions to collaborate with institutions in their home nation and on the same continent.

As shown in Table 2 and Fig. 4, the productive authors were mainly from Asian, European and North American countries. Christie M Ballantyne and Peter Libby whose scholarly contributions occupied an eminent position in the field were probable initiators of collaborative relationships. Even though Wolfgang Koenig (n = 21) and Oliver Soehnlein (n = 18) did not rank among the top ten, their high betweenness centrality of 0.13 indicated they had published potentially revolutionary material and were actively engaged in collaborative research worldwide.

In Table 3, literature on this field was largely published in journals from Western countries. In general, top prolific journals were distributed in Q1 or Q2, suggesting that high-quality and well-designed studies constituted the evidence base for ASCVD and inflammation research.

Journals with high co-citations are referred to as mainstream journals, to which researchers are dedicating great attention. There were a high number of co-citations in journals with high IFs and in Q1 journals, which indicates that top-tier journals benefited from consistent interest from scholars. In addition, the journal with the highest production and the most co-citations included *PloS one*, *Atherosclerosis*, *Arteriosclerosis, thrombosis, and vascular biology*, *Stroke*, and *Circulation research*, which were deemed core journals in the field in that a heightened interest in certain topics reported by these journals may influence the research foci, and we might include these journals when tracking research progress, given the volume of publications they produce.

### 4.2 Intellectual Base

Co-cited publications represent the frequency with which two publications are referenced by another, and can be viewed as a knowledge base related to specific subject matter. As shown in Table 4, five articles from *The New England journal of medicine*, two from *Nature*, one from *Nature medicine*, one from *Nature genetics*, and one from *Lancet* were identified to constitute the intellectual base of ASCVD and inflammation research. Besides, the topics covered by the top co-cited articles with the strongest betweenness centrality shown in Table 5 were key components in the knowledge structure of this field.

### 4.3 Hot Topics and Frontiers

The burst of citations within a subject indicates emerging trends within that field [103]. In Fig. 7, among the top 25 references, six references whose citation bursts continued to 2022 have attracted considerable attention from the scientific community, thus reflecting the hot topics in the field [66, 67, 104, 105, 106, 107].

In Fig. 11, the keywords with ongoing citation bursts (i.e., ongoing sharp increases in citation counts) were identified to explore the hot themes in this field. These topics are not distinct, but interrelated and influence one another, so we highlight their common aspects in order to illuminate the hot issues and make them more focused.

#### 4.3.1 NLRP3 Inflammasome

As a chronic inflammatory condition, atherosclerosis features lipid deposition, leukocyte infiltration, and VSMC proliferation. An inflammatory environment in the plaque is decisively controlled by the activation of atheroma macrophages.

In early atherosclerosis, macrophage-derived NLRP3 inflammasome suggests beneficial effects on plaque stability, which are mediated by their involvement in inflammatory anti-injury reaction. NLRP3 inflammasome activation in late atherosclerosis causes macrophage death and a significant amount of lipid release, both of which increase plaque vulnerability [108, 109]. NLRP3 inflammasome activation can result from extracellular cholesterol crystal uptake, leading to lysosomal damage and the accumulation of cholesterol in the plasma membrane. Mice treated with NLRP3 inhibitors or NLRP3 genetic deletions suffer less atherosclerosis [110, 111].

The NLRP3 inflammasome is activated in two steps, first by priming and then by activating, which allows caspase 1 to release IL-1β and IL-18 to mature forms. In fact, a variety of cell types can produce IL-6 when they are stimulated by IL-1β or IL-18. A characteristic shift towards an acute phase pattern thus occurs in hepatocytes due to a change in protein synthesis pattern, which amplifies the inflammatory cascade inside the vessel [112].

Because the NLRP3 inflammasome sequelae are critical to the development of atherosclerosis by orchestrating the expression of inflammatory cytokines, it is likely that direct inhibition of inflammatory pathways through the NLRP3 inflammasome would be a promising therapy for atherosclerosis. At a molecular level, evidence suggests that autophagy, an intracellular degradation system that keeps cells in a state of homeostasis, down-regulates the activation of NLRP3 inflammasome [113]. A wide range of stimuli, including reactive oxygen species (ROS) from mitochondria, activate NLRP3 inflammasome, and autophagy inhibits their activation by the removal of damaged mitochondria [114]. Besides, The autophagic process inhibits inflammasome activity by degrading NLRP3 inflammasome via ubiquitination and modulating its activity. In fact, an autophagy defect in Atg $5^{\text{-/-}}$ mice is reflected in accelerated atherosclerosis and increased NLRP3 inflammasome activity. Furthermore, the phosphorylation of protein kinase A (PKA) is known to negatively regulate NLRP3 inflammasome. The G protein-coupled bile acid receptor (GP-BAR1) (also known as TGR5) activates PKA, resulting in the ubiquitination of NLRP3, which can be correlated with the phosphorylation of NLRP3. It has been shown that TGR5 can reduce the progression of atherosclerosis by promoting cholesterol efflux and reducing inflammatory reactions; however, their anti-inflammatory mechanisms remain to be fully clarified. The mechanisms by which NLRP3 inflammasome inhibits atherosclerosis also requires further studies [115, 116].

Results from the CANTOS and Cardiovascular Inflammation Reduction Trial suggest that the inhibition of the inflammasome through the CRP pathway lowers vascular risk [117]. Therefore, the NLRP3 inflammasome has attracted interest as a target in atherosclerosis due to its ability to generate both active forms of IL-1β and IL-18. Nevertheless, further safety studies are needed for novel NLRP3-inflammasome inhibitors since such systemic inhibition of inflammatory pathways is likely to interact with immune homeostasis in ways that pose potential risks. Also, there is currently no data available to determine whether targeting IL-6, downstream of IL-1β and IL-18, is a better option, which might theoretically mute inflammation with less impairment of host defenses [118].

#### 4.3.2 Gut Microbiota and TMAO

Evidence is mounting that gut microflora plays a critical role in mucosal integrity and tolerance. Alterations in the gut microbiota may trigger an inflammatory state in the gut, which could lead to systemic inflammation. Dietary fibers are fermented in the gut microbiota, resulting in SCFAs, which are the main energy source for colonocytes, and they are presumably involved in colon epithelium renewal [119]. The decreased production of SCFAs that results from low dietary fiber consumption increases epithelial permeability by impairing epithelial metabolism. Therefore, either local or systemic inflammation may ensue as a result of the translocation of bacteria and LPS created by gut bacteria. Specifically, LPS binds to the TLR4 complex, along with the co-receptor CD14, which activates myeloid differentiation factor 88/NF-NF-κB pathway, resulting in increased production of pro-inflammatory cytokines including IL-6, IL-1, IL-27, and TNF-α, all of which are involved in atherosclerosis development [120]. Karlsson *et al*. [121] compared the gut microbiomes of atherosclerosis patients with control samples by functional characterization, and found that butyrate-acetoacetate CoA-transferase which originates from Clostridium sp. SS2/1 correlated negatively with high-sensitivity CRP in blood. In fact, butyrate is found to increase the generation of anti-inflammatory Treg cells and reduce inflammation in the colon [122].

Furthermore, the role of TMAO in accelerating atherosclerosis has recently received attention. After ingestion, bacteria residing in the gut convert L-carnitine and choline into trimethylamine, which is absorbed and converted by hepatic flavin monooxygenases to TMAO. The TMAO increases macrophage uptake of LDL-cholesterol and speeds foam cell formation by activating macrophage scavenger receptors CD36 and steroid receptor RNA activator 1, which explains its pro-atherogenic effects [123]. In the gut, TMAO precursors can be attenuated by administering poorly absorbed broad-spectrum antibiotics [123, 124]. TMAO further promotes atherosclerosis by activating the CD36-dependent MAPK/Janus kinases (JAKs) pathway and triggering the expression of VCAM-1, TNF-α and IL-1β via the NF-κB pathway [125, 126]. In addition, researchers have reported that TMAO causes hyper-reactivity in platelets, which can facilitate thrombosis, resulting in athero-thrombotic events [127].

In humans, circulating TMAO levels are associated with coronary artery disease burden and mortality in coronary artery disease patients in a dose-dependent fashion [128, 129]. The consumption of resveratrol can increase the ratio of *Bacteroidetes* to *Firmicutes* and the growth of *Bacteroides*, *Lactobacillus*, and *Bifidobacterium*, which have been shown to reduce levels of TMAO [130]. The possibility of modulating TMAO-producing bacteria to lower plasma TMAO levels sounds intriguing, but an effective treatment mode has not yet been identified.

Though TMAO appears to contribute to atherosclerosis in a variety of ways, the causal effect of TMAO on atherosclerosis is still being explored. In addition, even some studies have reported results contrary to the studies mentioning TMAO’s effect on atherosclerosis. In the report from Bäckhed laboratory, germ-free mice and conventionally raised Apolipoprotein E deficient (*${\mathrm{Apoe}}^{\text{-/-}}$*) mice were fed either a chow or a Western type diet (WTD) containing standard low levels of choline (0.08%) or enriched in choline (1%–1.2%) [131]. As expected, in germ-free mice fed with chow diet or WTD, TMAO was barely detectable, and supplementation with choline did not increase plasma TMAO in germ-free mice, while conventionally raised mice showed an increase in plasma TMAO after supplementation with choline [131]. It is interesting to note that germ-free mice fed with chow diet had greater aortic root lesions than conventionally raised mice fed with chow diet; however, germ-free and conventionally raised mice fed with WTD had no difference in aortic lesions [131]. In mice fed WTD, the absence of microbiota had no effect on atherosclerosis [131]. In conventionally raised male mice, elevated plasma TMAO had no effect on atherosclerosis and associated lesions [131]. It was concluded that gut microbiota influences atherosclerosis in a dietary-dependent manner and is associated with plasma cholesterol levels [131]. Therefore, TMAO’s effects on atherosclerosis are not completely understood. Also, it is unclear how useful it is to measure TMAO levels in clinical scenarios, especially as there are no treatments to lower TMAO levels beyond lifestyle modifications in clinical practice.

Overall, the microbiota has been studied mostly through cross-sectional studies to date in relation to low-grade inflammation and cardiovascular disease. We need to conduct further prospective studies to confirm these findings. With respect to how the gut microbiota can be regulated and how to break the possible feedback loop between gut inflammation and systemic inflammation, a strategy that employs prebiotics, probiotics, and natural products to promote beneficial changes in the intestinal flora and to improve epithelial integrity seems promising.

#### 4.3.3 Autophagy

The major atherosclerotic plaque cells present in the fibrous cap and around the necrotic core (e.g., ECs, VSMCs, macrophages) undergo autophagy. The role of EC autophagy in atherosclerosis progression is still debated. One study shows that oxidized LDL (ox-LDL) increases the von Willebrand factor and P-selectin secretion in ECs, indicating that this may be a mechanism through which ox-LDL inhibits the sirtuin-1/forkhead box transcription factor O1 pathway and increases autophagic flux in ECs. Hence, reducing arterial thrombosis and atherosclerosis might be possible by increasing autophagic flux [132]. Endothelial autophagy also inhibits vascular inflammation through the down-regulation of ICAM-1, VCAM-1, E-selectin, and NF-κB signaling [133]. Induction of autophagy in TNF-stimulated human umbilical vein ECs (HUVECs) reduces the expression of inflammatory cytokines via activation of the PKA/adenosine 5’-monophosphate (AMP)-activated protein kinase (AMPK)/SIRT1 pathway [134]. Other studies, however, have shown that EC autophagy promotes atherosclerosis. Autophagic death induced by excessive autophagy in ECs may destroy the stability of atherosclerotic plaques [135]. In addition, stress-induced overactivated autophagy increases apoptosis in ECs, while recombinant thrombomodulin treatment can inhibit EC apoptosis by modulating rapamycin (mTOR)-dependent autophagy [136].

As for VSMC autophagy in atherogenesis, a study by Pi S *et al*. [137] demonstrated that the activation of the P2RY12 receptor triggered mTOR through the phosphatidylinositol 3-kinase/Akt pathway, resulting in the inhibition of autophagy in advanced atherosclerosis and reduced cholesterol outflow. However, miR-223 inhibited the formation of foam cells via inducing the autophagy of VSMCs [138]. According to another study, ox-LDL induced atherogenesis is inhibited by AMPK/mTOR signaling, which increases autophagy [139]. The knockdown of sterol regulatory element-binding cleavage-activating proteins in VSMCs of *${\mathrm{ApoE}}^{\text{-/-}}$* mice reduces lipid accumulation and oxidative stress as well as stimulates VSMC autophagy via ROS/AMPK pathways [140]. Nevertheless, in VSMCs, tissue-specific deletion of Atg7 causes accumulation of SQSTM1/p62 and accelerates stress-induced premature senescence [141]. In this way, senescent VSMCs develop an inflammatory, senescence-associated secretory phenotype driven by IL-1α that may contribute to atherosclerosis [142]. Similarly, VSMCs that undergo excessive autophagy destabilize plaques by reducing collagen synthesis, resulting in a thinner fibrous cap [143].

The autophagic process is closely linked to the formation and development of atherosclerotic plaques composed of macrophage-derived foam cells. By selectively inhibiting the PI3K/Akt/mTOR pathway, autophagy is activated in macrophages, improving atherosclerotic plaque stability [144]. By degrading the NLRP3 and ASC subunit of the inflammasome, macrophage autophagy is shown to inhibit inflammation in ox-LDL-induced foam cells [145]. By contrast, atherosclerosis occurs and is exacerbated by impaired or defective autophagy in macrophages. Defects in macrophage ATG5 promote apoptosis and oxidative stress in macrophages, and worsen lesional efferocytosis in advanced atherosclerosis [146]. Therefore, activating macrophage autophagy may offer new therapeutic approaches to treat atherosclerosis.

The above results show different autophagy levels in different cells, which can be manifested as defective, basal, mild or over-induced autophagy, accompanies the entire process of atherogenesis. A growing body of evidence suggests that while the basic or moderate form of autophagy suppresses inflammation, increases cell survival, and protects against the progression of atherosclerotic plaques, the impaired or excessive form promotes inflammation, and accelerates cell death, and apoptosis, further exacerbating plaque instability and rupture. As such, targeting atherosclerotic plaque cell autophagy could be a viable approach for atherosclerosis treatment, which aims to control autophagy without causing harmful consequences.

#### 4.3.4 LncRNAs

Several atherogenic processes may be influenced by lncRNAs, which are defined as non-protein-coding transcripts longer than 200 nucleotides. By regulating autophagic flux, lncRNAs act as molecular switches that regulate lipid metabolism and the inflammatory response in the vasculature despite their poor conservation between species. This opened up the field for the investigation into relationships among lncRNAs, autophagy and atherosclerosis.

For example, through binding mixed lineage kinase domain-like protein (MLKL) promoter, lncRNA FA2H-2 regulates autophagy and inflammation in atherosclerosis. After the suppression of MLKL expression in SMCs and ECs in response to ox-LDL, autophagic flux is enhanced and inflammation is diminished [147]. The reduction of FA2H-2 expression in VSMCs and ECs leads to a significant increase in the expression of MLKL, suppresses autophagic flux, and induces the expression of IL-1β, IL-6, and IL-8, and TNF-α [147]. FA2H-2 promotes autophagy in VSMCs and ECs, thus reducing inflammation by acting as an atheroprotector.

MALAT1 is another interesting lncRNA under investigation. Recently, a study demonstrated that MALAT1 possesses anti-inflammatory properties in part through its binding to miR-503 [148]. Mechanistically, atherosclerotic plaque formation is mitigated by MALT1 by blocking the adhesion of myeloid cells to ECs and reducing pro-inflammatory cytokine production [148].

Overall, lncRNAs, as a regulator, may have cell-type-specific expression patterns, and are appealing pharmacological targets. In order to develop therapeutic strategies targeting lncRNAs for atherosclerosis, it is essential to unearth how lncRNA-mediated autophagy is regulated in atherosclerotic plaque cells.

#### 4.3.5 Exosomes

The present study also identified EVs, especially exosomes as another emerging topic in the field. The extracellular vesicles called exosomes are nanosized with a diameter of 30 to 150 nm and contain the RNA, DNA, proteins, and lipids from the donor cells [149]. Exosomes can interfere with the function of ECs, VSMCs, and macrophages, causing either pro-atherosclerosis or anti-atherosclerosis, depending on the donor cells’ condition.

For example, exosomes secreted by ox-LDL-stimulated THP-1 monocyte ferrying lncRNA LIPCAR, miR-106a-3p, and GAS5 are reported to promote atherosclerosis by altering the phenotypes of ECs and VSMCs [150, 151, 152]. By contrast, exosomal miRNAs derived from mesenchymal stem cells, bone marrow-derived macrophages, as well as platelets may exert anti-atherosclerotic effects [153, 154, 155]. In addition, although low levels of lncRNAs are present in exosomes, increasing data suggest that exosomal lncRNAs are new clues to the pathogenesis of ASCVD and more attention is needed to help clarify the possible relationships among exosomes, exosomal lncRNAs and miRNAs [156].

#### 4.3.6 Nrf-2

The Nrf-2 protein encoded by the *NFE2L2* gene has been closely linked with atherosclerosis, yet its role seems antagonistic, preventing as well as promoting its development. Depletion of Nrf2 in bone marrow-derived cells is shown to attenuate the formation of atherosclerotic plaques, whose mechanism probably implicates the reduction in pro-inflammatory M1 macrophage [157]. The Nrf2-mediated role in potentiating atherosclerosis also involves NLRP3 inflammasome activation, decreased uptake of acetylated LDL, and up-regulated expression of CD36 scavenger receptor in macrophages [158, 159, 160, 161].

By contrast, the activation of Nrf2/HO-1 signaling is shown to decrease gene expressions of inflammatory factors such as TNF-α, IL-1β and IL-6 in LPS-stimulated RAW264.7 cells, which highlights the anti-inflammatory activity of Nrf2 [162]. Hu Q *et al*. [163] has found that dihydromyricetin pre-treatment suppresses pyroptotic cell death and reduces IL-1β release by the activation of the Nrf2 signaling to inhibit NLRP3 inflammasome activation in palmitic acid-induced HUVECs. In addition, Nrf2/HO-1 signaling is a popularly researched pathway through whose activation natural extracts protect ECs from injury in atherosclerosis [164].

Thus, *in vitro* and *in vivo* experiments with Nrf2 in atherosclerosis are, however, ambiguous and heavily dependent on the atherosclerotic lesion stage or animal model. Also, further research is needed to better define its contribution to human atherosclerosis.

#### 4.3.7 Reasons that Contribute to These Hot Research Topics 

The reasons for these foregoing topics being research hotspots are multifaceted either because these targets began to emerge in the most recent years or because the lack of suitable methodologies in earlier studies have made it challenging to investigate their involvement in ASCVD.

As a result of advances in biochemistry, cell biology, and genetic engineering, experimental atherosclerosis has experienced a flurry of activity, leading to thousands of experimental papers that shed light on diverse aspects of inflammation in the regulation of ASCVD. In spite of these impressive experimental results, a gap remained between the lab and the clinic.

In experimental aspect, despite the availability of *${\mathrm{ApoE}}^{\text{-/-}}$* and the LDL receptor-deficient (*${\mathrm{LDLr}}^{\text{-/-}}$*) mice, studies were conducted using mice to investigate the effects of the potent proinflammatory cytokines, IL-1β, and IL-18 on atherosclerosis in the early 2000s [165, 166]. It was not until nearly ten years later that Duewell P *et al*. [110] published their landmark study that showed atherosclerotic lesions in *ApoE* deficient mice on a high-fat diet contained cholesterol crystals that increased as the disease progressed. In particular, these crystals were observed to co-localize with NLRP3 inflammasomes and promoted the release of IL-1β [110]. Further, it was shown that *${\mathrm{LDLr}}^{\text{-/-}}$* mice that received bone marrow transplants with NLRP3-deficient, ASC-deficient, and IL-1α/β-deficient bone marrow showed markedly reduced atherosclerosis [110]. Due to the cross-talk between inflammation and lipid metabolism that the NLRP3 inflammasome mediates, intensive studies have been been spotlighted on the role of the NLRP3 inflammasome in atherogenesis in mouse models, including its activation in ECs, immune cells (such as monocytes, macrophages and dendritic cells), and SMCs as well as its mechanism of action in atherosclerotic disease since then. In spite of the proliferation of NLRP3 inflammasome research topics in basic science, its clinical application seems stagnant as few biomarkers of inflammation have moved from the lab to the clinical setting.

With regard to clinical research, also in the early 2000s, lipid-lowering trials demonstrated that statin therapy reduced the level of circulating hsCRP, which confirmed at least partially the interaction between inflammation and lipid metabolism [167, 168, 169]. It was identified in landmark statin trials that patients who demonstrated reductions in LDL-cholesterol to less than 70 mg/dL as well as a reduction in hsCRP to less than 2 mg/L had a greater cardiovascular benefit than those with only a significant reduction in LDL-cholesterol levels [170, 171, 172]. A consequence of this has led to the concept of residual inflammatory risk, defined as persistently elevated hsCRP despite adequate atherogenic lipid lowering, a phenomenon seen in 30%–40% of all statin trial participants [173].

Observational studies have shown strong associations between CRP levels, coronary atherosclerosis, and vascular risk [174, 175]. In spite of the fact that CRP does not directly contribute to atherothrombosis [176], its application as a biomarker of inflammation has begun to bridge the gap between experimental and clinical findings. In the decade that have followed the initial investigation of CRP in cardiovascular disease, dozens of studies have accumulated evidence that strongly associates inflammation as measured by CRP with a number of outcomes associated with a range of cardiovascular outcomes [177]. As far as other biomarkers of inflammation and oxidative stress are concerned, none have proven to be as reproducible and clinically practical as CRP measured with the highly sensitive method [178].

Most importantly, CRP integrates inflammatory signals generated by a variety of sources. As previously mentioned, while statin therapy, such as rosuvastatin, may promote some of its clinical benefit through direct anti-inflammatory effects, because of concomitant reduction in LDL [179], it is not possible to determine rigorously the extent to which this agent’s anti-inflammatory effects contribute to the clinical benefit observed, independent of LDL lowering.

With the identification the canonical pathway that links the NLRP3 inflammasome with IL-1/IL-6/CRP [180], targeting the NLRP3 inflammasome may provide therapeutic benefits in ASCVD, especially in light of the results of clinical trials such as CANTOS, which demonstrated that inhibiting inhibiting IL-1β, a main product of NLRP3 inflammasome activation, diminished clinical endpoints in patients with atherosclerotic disease independent of lipid level lowering, accompanied by a reduction in IL-6 and CRP [66, 181]. Also, methotrexate failed to inhibit IL-6/IL-1β pathway and acted via the inhibition of aminoimidazole-4-carboximaide ribonucleotide, resulting in subsequent elevations in adenosine levels, and no reduction in cardiovascular endpoints [182], further suggesting inflammation that leads to atherogenesis is pathway-specific rather than generalized. For all these reasons, it appears the NLRP3 inflammasome is linked to ASCVD and understanding the mechanisms regulating NLRP3 inflammasome activation and subsequent signaling is vital to gain insight into potential therapeutic strategies to prevent ASCVD, which makes NLRP3 inflammasome a research hotspot in this field.

With regard to the research on gut microbiota, the atherosclerotic plaque has been found to contain a number of bacteria, including *Streptococcus*, *Pseudomonas*, *Klebsiella*, *Veillonella* spp., and *Chlamydia pneumoniae, *making it a microbial environment on itself [183, 184, 185]. Most studies, however, failed to link plaque microbiota composition with outcomes such as plaque vulnerability, rupture, or cardiovascular events [186]. In addition, antibiotic treatment as secondary prevention, which seeks to eliminate plaque microbiota, did not result in a reduced incidence of cardiovascular events [187]. As a result, these studies failed to provide evidence in support of the causal role of direct vessel wall infection in plaque formation. In contrast, the hypothesis that distant infections can elicit an auto-immune inflammatory response through molecular mimicry appears to be more plausible [188].

Since the development of 16S rRNA gene amplicon sequencing and shotgun metagenomic sequencing, we have therefore received a greater understanding of the role of gut microbiota in ASCVD [189]. Specifically, metagenomic sequencing allows not only species-level resolution of compositional data, but also enables the assessment of differences in gut microbiota functionality, since compositional differences do not always translate into functional differences. Human cross-sectional studies found that patients with symptomatic atherosclerosis have higher abundances of *Collinsella* genus, *Enterobacteriaceae*, *Streptococcaceae*, and *Klebsiella*species, as well as lower abundances of SCFA-producing bacteria *Eubacterium*, *Roseburia*, and *Ruminococcaceae *spp. as compared with healthy controls in the gut microbiota [121, 190, 191]. In addition, with the introduction of metabolomics approach, it has been shown that plasma levels of TMAO and its precursors, choline and betaine has been shown to predict the risk of cardiovascular disease [124].

The advent of culture-independent sequencing technologies and omics led to a huge amount of associative data, but these data are not robust to causal investigation, despite their usefulness. Further, in animal studies, fecal microbiota transplantation (FMT) provides causal evidence that gut microbiota composition is associated with atherosclerosis. For instance, mice transplanted with a microbiota composition that was more pro-inflammatory from *${\mathrm{Caspase1}}^{\text{-/-}}$* mice had 29% larger plaques than those in the control group [192]. In this regard, gut microbes could indirectly contribute to atherosclerosis by producing pro-atherogenic metabolites.

Overall, the spotlight on the role of gut microbiota and metabolites such as TMAO in atherosclerosis benefits both from the advancement in sequencing technologies, bioinformatics tools, and omics technologies, and the efforts to determine whether microbial changes are driving disease states or rather being driven by them through the introduction of emerging methodological approach (e.g., FMT).

Over 50 years ago, autophagy was identified as a mechanism that sequesters and degrades cytosolic components via the lysosome pathway; however, the role of autophagy in atherosclerotic lesions remains unappreciated due to incompletely characterized ultrastructural features. The first hint for its contribution in atherosclerosis came from the paper by Perrotta I [193] in 2013, in which transmission electron microscopy revealed autophagy in all primary cell types (i.e., macrophages, VSMCs, and ECs) in human atherosclerotic plaques. Since then, a growing number of studies have provided further evidence that autophagy occurs in atherosclerosis with the expression of autophagy marker, such as microtubule-associated protein light chain 3-II and microtubule-associated protein 1 light chain 3 detected in various cells in unstable atherosclerotic plaques [194].

Therefore, the cell-specific contributions of autophagy to atherosclerosis initiation, progression, and regression were investigated. In addition, with the discovery of other forms of regulated cell death such as pyroptosis and ferroptosis in recent years [195], their intricate overlapping effects and interactions have further increased interest in autophagy as a hot topic in atherosclerosis research.

The role of exosomes in cardiovascular diseases has, as evidenced by a bibliometric analysis, emerged as a “star target” only in the last decade; especially in the past five years, the area has gained significant momentum [196]. Exosomes are crucial mediators of intercellular communication during atherosclerosis development, as described above; a major obstacle to the reaseach on exosomes is the isolation methods, since obtaining high-purity exosomes is crucial for further research.

While there is currently no standardized method for the isolation of exosomes, many techniques have been developed based on the biochemical and physicochemical characteristics of exosomes, including differential ultracentrifugation, immunoaffinity capture, polymer-based precipitation, ultrafiltration, and size exclusion chromatography, each of which has its own advantages and disadvantages [197, 198]. These significant methodological advances have resulted in further insights into the role that intercellular communication plays in ASCVD pathogenesis, thereby leading to exosomes as an emerging mediator of atherogenesis. It is expected that with the introduction of promising exosome isolation methods very recently, such as ExoTIC, acoustofluidic platform, and alternating current electrokinetic microarray chip devices [199, 200, 201], further interest will be fueled in exosomal research, allowing them to be used in clinical settings for diagnostic or therapeutic purposes.

Non-coding RNAs (ncRNAs) were initially regarded as transcriptional noise or residual waste generated during the processing of RNA. Nevertheless, research interest expanded to miRNA in early 2000, with subsequent studies showing that miRNAs play a significant role in physiological processes and pathological outcomes. In fact, the first evidence for a putative role of ncRNA in vascular disease came from genome-wide association studies that identified the most significantly associated locus with coronary artery disease on the human chromosome 9p21 [202]. The region is adjacent to INK4 locus that encodes a lncRNA named ANRIL, also known as CDKN2BAS [203]. As opposed to miRNAs, whose biosynthesis and biological activities are well explored, lncRNAs are more heterogeneous and difficult to characterize. It is not easy to infer the specific function of lncRNAs from their sequence or structure, unlike microRNAs or proteins.

However, the field of lncRNAs has benefited from the advance in genomic technologies, including the availability of fast and cost-effective sequencing technologies and computational resources, with a new class of lncRNAs discovered and annotated every year. In this regard, lncRNAs have been shown to be expressed in major types of cells in atherosclerotic lesions and are implicated in several atherogenic processes [204].

Furthermore, recent studies have concentrated on the characterization of molecules within exosomes, as well as their use as diagnostic agents. For example, exosomal lncRNA HIF1A-AS1 was found by Wang Y *et al*. [205] to be a potential biomarker for atherosclerosis. In this way, by rapidly advancing technologies and protocols for isolating, purifying, and detecting exosomes, in addition to rapidly developing genomic tools, exosomal lncRNAs are also positioned to undergo clinical translation.

Since Nrf2 was first cloned in 1994, the field of Nrf2 is relatively young. It was in the mid- to late 1990s, when the first *${\mathrm{Nrf2}}^{\text{-/-}}$* mouse was generated [206], that many of the studies were conducted on molecular interactions that drive Nrf2 signaling. Moreover, the *${\mathrm{Nrf2}}^{\text{-/-}}$* mouse provided insight into Nrf2’s role in chemoprevention as oltipraz, a dithiolethione, lost its chemoprotective effects in mice deficient in Nrf2 [207]. Upon revealing that Nrf2 plays a chemoprotective role, the focus shifted toward identifying methods for activating the Nrf2 pathway.

As recently as 2006, however, the discovery that KEAP1 mutations that leads to chronically elevated levels of Nrf2 was found in non-small lung cell carcinomas presented the first evidence that Nrf2 may contribute to cancer progression and chemoresistance [208], which was later referred to as the “dark side” of Nrf2. Despite Nrf2’s known benefits, new research has revealed the previously unappreciated complexity of the Nrf2 signaling network, growing evidence that careful regulation of this pathway is crucial to disease prevention. In the field of atherosclerosis research, the same applies. Studies have demonstrated that Nrf2 plays a dual role in atherosclerosis, as previously described. The paradoxical role of Nrf2 in atherosclerosis thereby spurs further research into its role in the various stages of plaque progression before it is considered a new therapeutic target due to the relative lack of understanding regarding its complex and diverse mechanisms.

## 5. Conclusions

The bibliometric profile of ASCVD and inflammation in the last decade aims to identify, evaluate and to visualize all literature published regarding qualitative, qualitative and chronological aspects. The emergence of a variety of experimental and clinical observations points to chronic inflammation as a key factor in ASCVD, thus sparking enthusiasm for this area of research. We demonstrated the leading position of the Asian, European and North American countries in the field in terms of quantitative, qualitative and collaborative parameters. In light of the complex mechanisms involved, identifying the causal pathway underlying inflammation and ASCVD is a challenging endeavor. Current research in the area has described the NLRP3 inflammasome, gut microbiota and TMAO, autophagy, lncRNAs, exosomes, and Nrf-2 as hot topics in the field, which may be promising directions in both basic and clinical research.

## Supplementary Material

Supplementary material associated with this article can be found, in the online version, at https://doi.org/10.31083/j.rcm2309317.
