# Supplementary figures and images for: Research Landscape on Atherosclerotic Cardiovascular Disease and Inflammation: A Bibliometric and Visualized Study
Source: Rev Cardiovasc Med. 2022 Sep 14;23(9):317. doi: 10.31083/j.rcm2309317 (PMC11262408; doi:10.31083/j.rcm2309317)

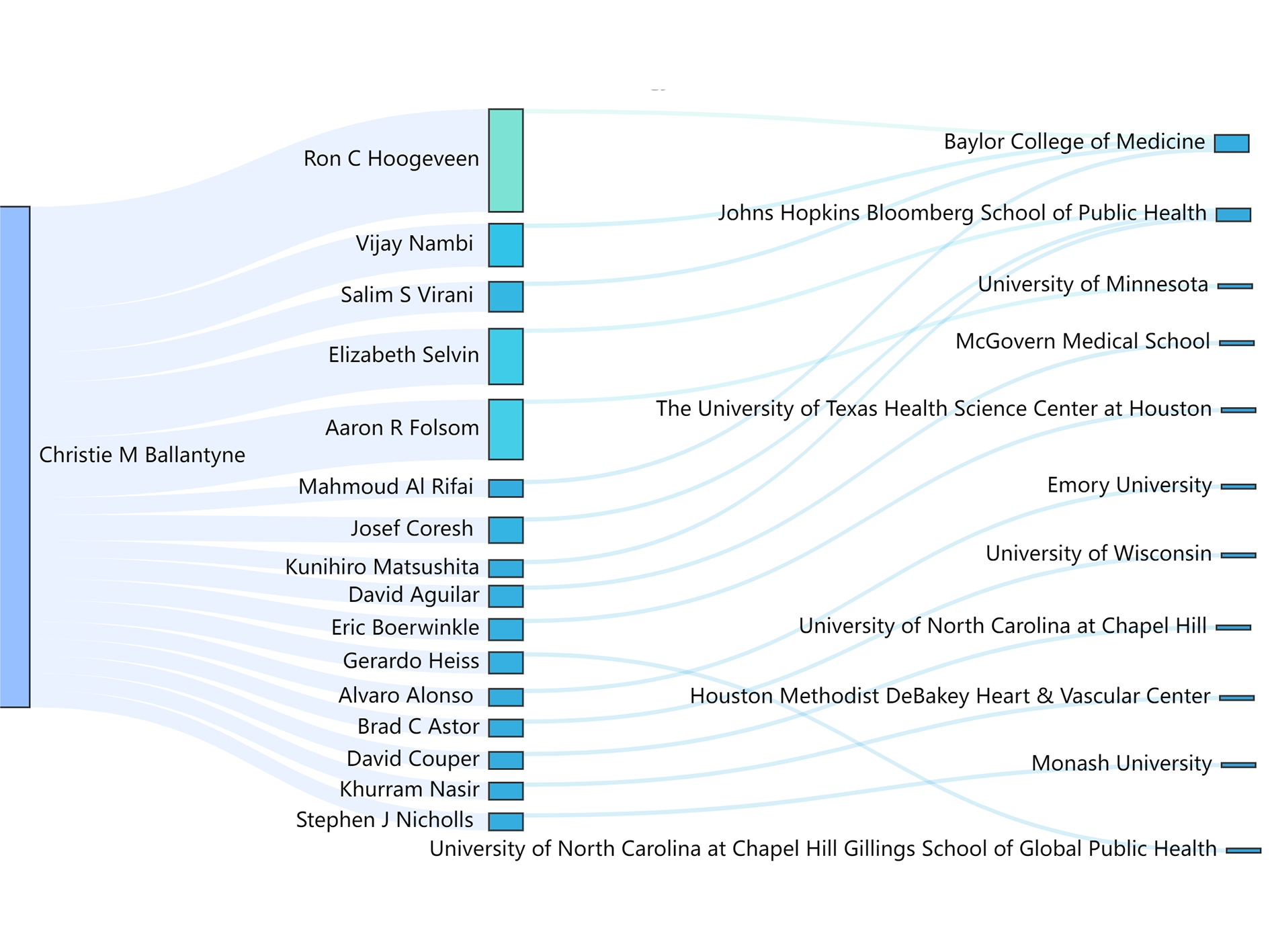

Supplement: Supplementary file 1 [file 2153-8174-23-9-317-s1.zip › 2153-8174-23-9-317-s1/Supplementary Fig. 1. The Sankey Plot representing the relationships among Christie M Ballantyne (Baylor College of Medicine), the collaborators, and the collaborators’ institutions.jpg]

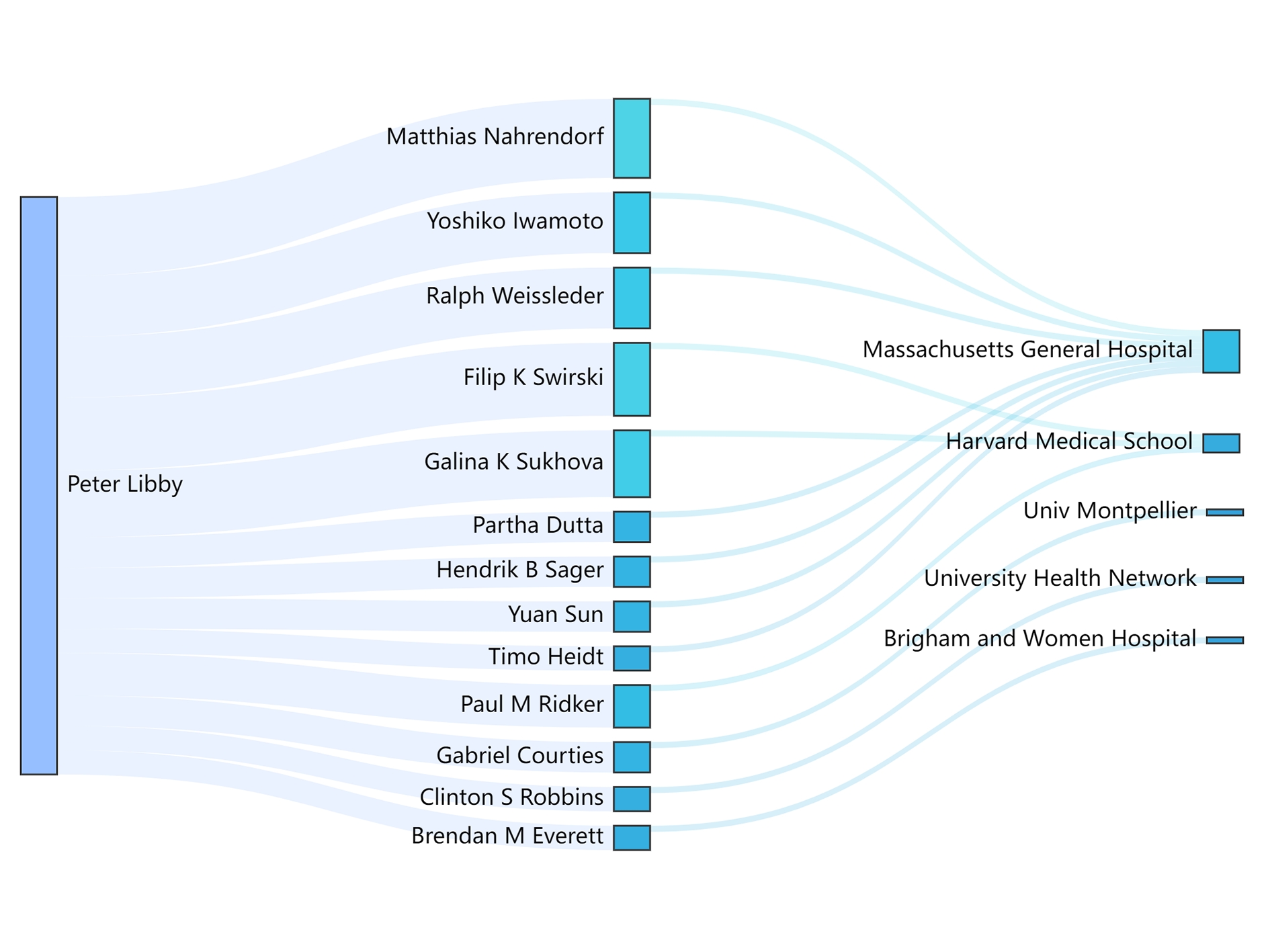

Supplement: Supplementary file 1 [file 2153-8174-23-9-317-s1.zip › 2153-8174-23-9-317-s1/Supplementary Fig. 2. The Sankey Plot representing the relationships among Peter Libby (Brigham and Women's Hospital, Harvard Medical School), the collaborators, and the collaborators’ institutions.jpg]

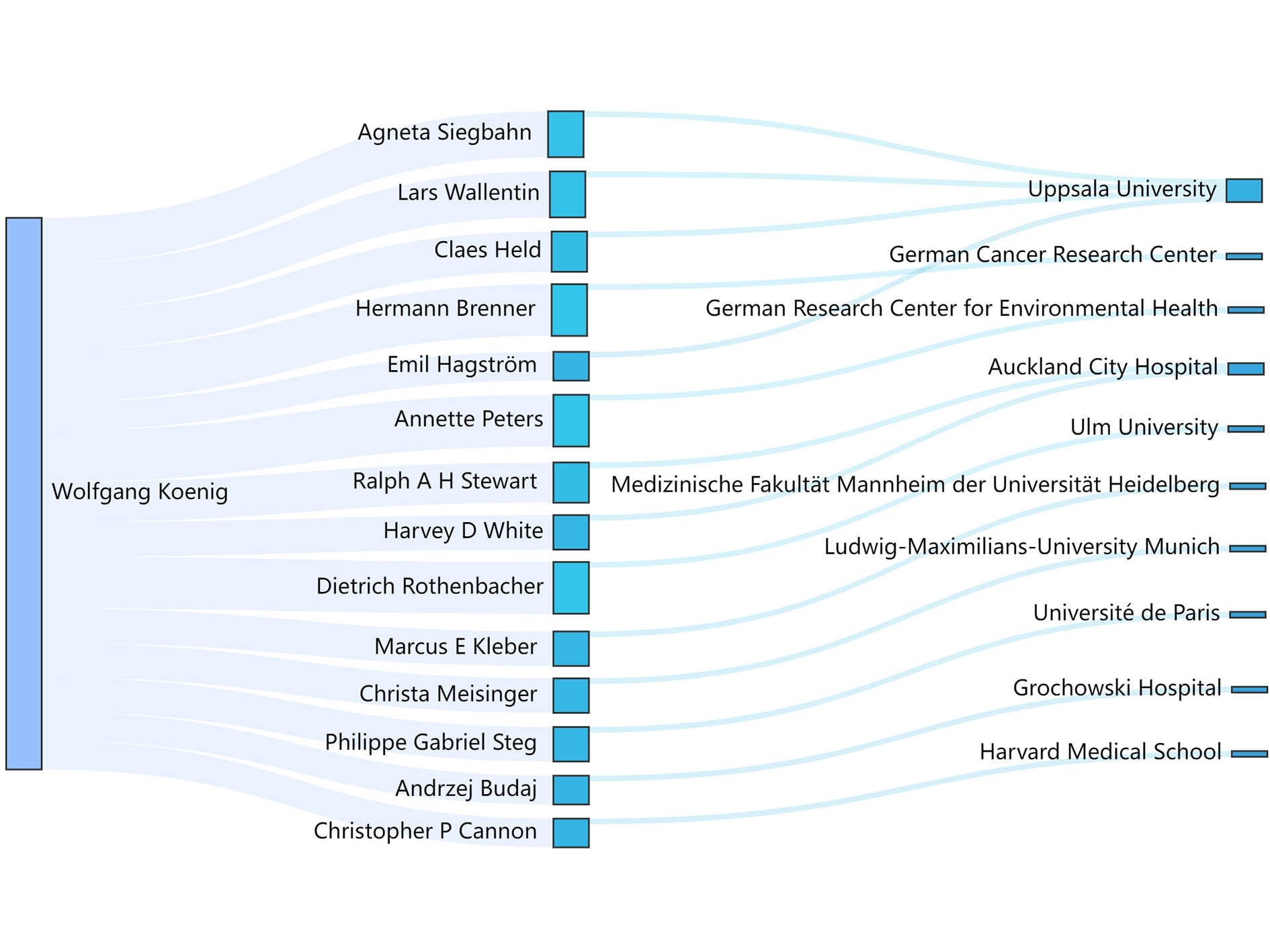

Supplement: Supplementary file 1 [file 2153-8174-23-9-317-s1.zip › 2153-8174-23-9-317-s1/Supplementary Fig. 3. The Sankey Plot representing the relationships among Wolfgang Koenig (Technical University of Munich, Ulm University), the collaborators, and the collaborators’ institutions.jpg]

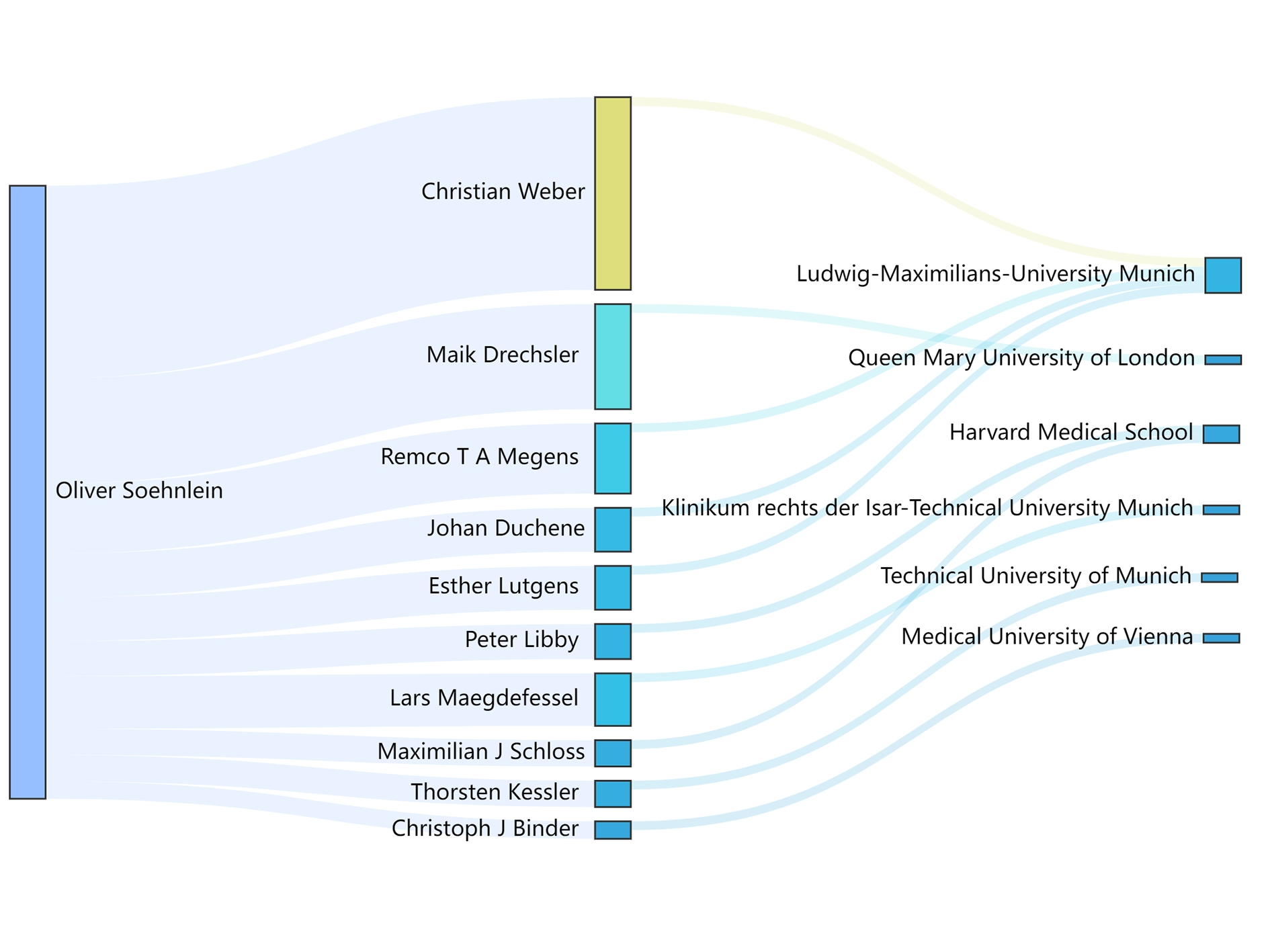

Supplement: Supplementary file 1 [file 2153-8174-23-9-317-s1.zip › 2153-8174-23-9-317-s1/Supplementary Fig. 4. The Sankey Plot representing the relationships among Oliver Soehnlein (Klinikum der Ludwig-Maximilians-Universität), the collaborators, and the collaborators’ institutions.jpg]
